# Supplementary material for: Extrusion fountains are hallmarks of chromosome organization emerging upon zygotic genome activation
Source: Nat Commun. 2026 Feb 14;17:2787. doi: 10.1038/s41467-026-69105-9 (PMC13018191; doi:10.1038/s41467-026-69105-9)
Supplement: Supplementary file 1 — Supplementary Information [file 41467_2026_69105_MOESM1_ESM.pdf]

Supplementary Information

for

"Extrusion fountains are hallmarks of chromosome organization emerging upon zygotic genome activation"

Aleksandra Galitsyna[*#1], Sergey V. Ulianov[*2,3], Mariia Bazarevich[4,5,6], Nikolai S. Bykov[7], Marina Veil[4], Meijiang Gao[4,5], Kristina Perevoschikova[8], Mikhail S. Gelfand[9], Sergey V. Razin[2,3], Leonid Mirny[†#1,10] and Daria Onichtchouk[†#,4,5,11]

*- these authors contributed equally to this work

†- these authors jointly supervised this research

# - corresponding authors:
galitsyn@mit.edu, leonid@mit.edu, daria.onichtchouk@biologie.uni-freiburg.de

1 - Institute for Medical Engineering and Science, Massachusetts Institute of Technology, Cambridge, MA, 02139, USA
2 - Institute of Gene Biology, Russian Academy of Sciences, 119334, Russia
3 - Faculty of Biology, M.V. Lomonosov Moscow State University, Moscow, 119991, Russia
4 - Department of Developmental Biology, University of Freiburg, Freiburg, 79104, Germany
5 - Signaling Research Centres BIOSS and CIBSS, Freiburg, 79104, Germany
6 - Spemann Graduate School of Biology and Medicine (SGBM), Freiburg, 79104, Germany
7 - Independent researcher, Moscow, Russia. Present address: Centro Nacional de Análisis Genómico (CNAG), Baldiri Reixac 4, Barcelona, 08028, Spain
8 - Faculty of Bioengineering and Bioinformatics, M.V. Lomonosov Moscow State University, Leninskiye Gory, 1, building 73, Moscow, 119991, Russia
9 - Independent researcher, P.O.Box 22, 125476, Moscow, Russia
10 - Department of Physics, Massachusetts Institute of Technology, Cambridge, MA, 02139, USA
11 - Koltzov Institute of Developmental Biology RAS, Moscow, 119991, Russia

Content

Supplementary Information ... **1**
Supplementary Figures ... **3**
Supplementary Methods ... **32**
Experimental model and subject details ... 32
Generation of MZsn, MZpn double mutant and MZtriple triple mutant embryos and maintenance of the mutant fish lines ... 32
Genomic DNA isolation and PCR for genotyping ... 33
Zebrafish sperm collection ... 33
Hi-C library preparation ... 33
Chromatin accessibility changes on fountains in MZtriple, MZspg, and MZnanog mutants compared to the wild-type ... 34
GREAT analysis ... 34
Replication timing data analysis ... 34
Hi-C data mapping ... 35
Hi-C data processing ... 35
Hi-C data visualization ... 35
Centromere positioning in Danio rerio ... 35
P(s) curves and derivatives ... 35
Removal of poorly mapped and surrounding genomic regions ... 36
Building developmental trajectories ... 36
Average Rabl configuration ... 36
Compartment calling and saddle plots ... 36
Insulation score ... 37
TAD calling ... 37
Fountain calling with fontanka ... 38
Enrichment of developmental regulatory elements at fountains ... 40
Hi-C snipping and average pileup ... 40
Differential fountains in MZtriple ... 40
ChIP-Seq data analysis ... 40
CTCF binding inference from ATAC-seq ... 41
Simulations of loop extrusion ... 41
1D simulation ... 41
3D simulation ... 42
In silico reconstruction of interaction probabilities ... 42
Goodness of fit of the simulations to real data ... 42
Parameter sweep ... 43
Supplementary Notes ... **44**
I. Fountain-like structures in other biological systems ... 44
II. Detailed characterization of zebrafish fountains ... 47
III. Limitations of fountain detection as an average pileup ... 49
IV. Realistic simulations of enhancer-targeted cohesin loading with CTCF barriers ... 50
V. Fountain calling in medaka and Xenopus ... 52
References ... **53**

Supplementary Figures

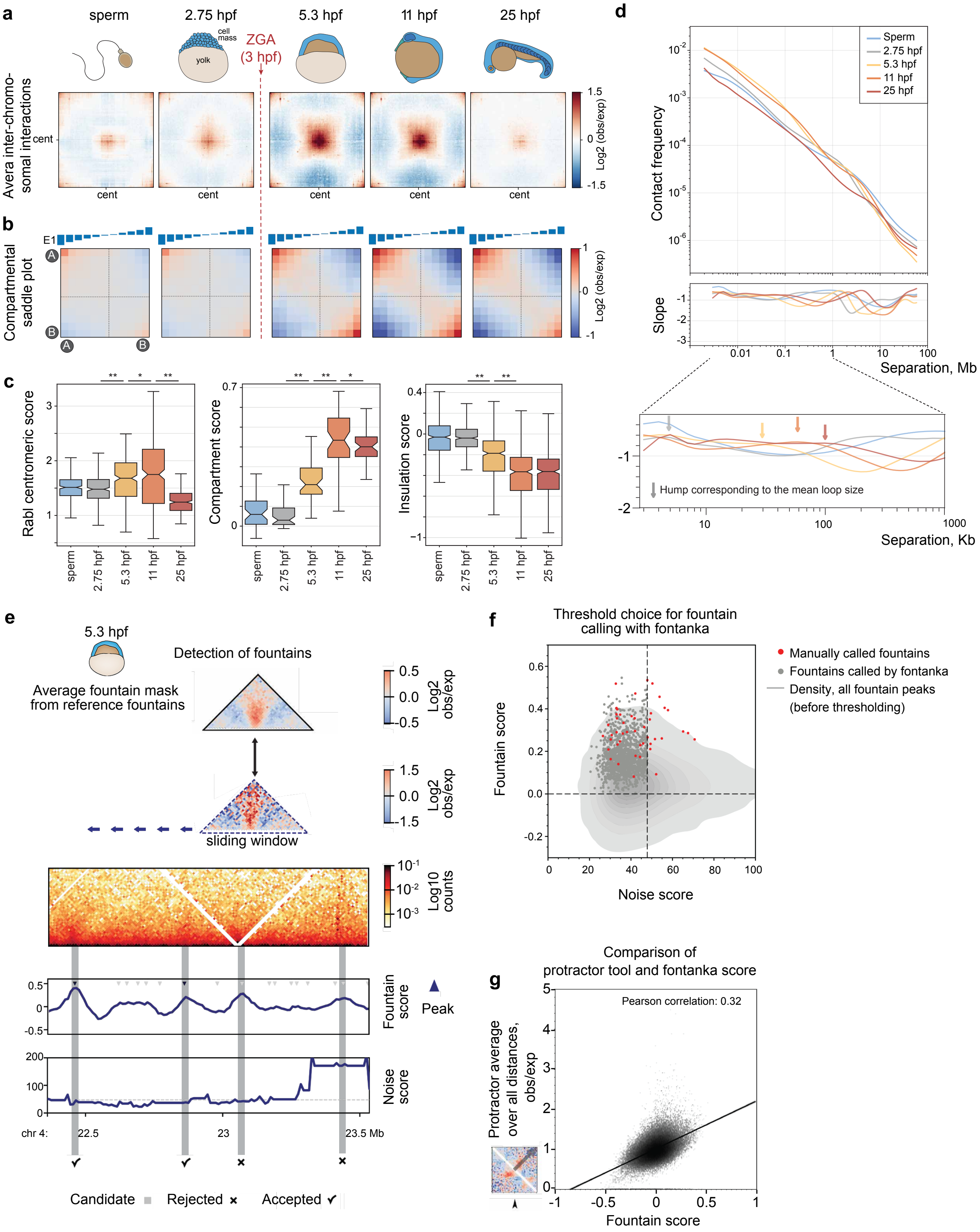

a
sperm
2.75 hpf
5.3 hpf
11 hpf
25 hpf
cell mass
yolk
ZGA (3 hpf)
Avera inter-chromo-somal interactions
cent
Log2 (obs/exp)
b
E1
Compartmental saddle plot
A
B
c
Rabl centromeric score
Compartment score
Insulation score
sperm
2.75 hpf
5.3 hpf
11 hpf
25 hpf
d
Contact frequency
Sperm
2.75 hpf
5.3 hpf
11 hpf
25 hpf
Slope
Separation, Mb
Hump corresponding to the mean loop size
Separation, Kb
e
5.3 hpf
Detection of fountains
Average fountain mask from reference fountains
Log2 obs/exp
sliding window
Log10 counts
Fountain score
Peak
Noise score
chr 4:
22.5
23
23.5 Mb
Candidate
Rejected
Accepted
f
Threshold choice for fountain calling with fontanka
Manually called fountains
Fountains called by fontanka
Density, all fountain peaks (before thresholding)
Fountain score
Noise score
g
Comparison of protractor tool and fontanka score
Pearson correlation: 0.32
Protractor average over all distances, obs/exp
Fountain score

Supplementary Figure 1.

Establishment of 3D chromatin organization and emergence of fountais in zebrafish development (related to Fig. 1).

Source data are provided as a Source Data file.

a. Average Rabl plots showing centromere-centromere interactions.

b. Saddle plots with compartmentalization.

c. Boxplots of Rabl centromeric scores (left), compartment strength (center), and insulation score (right) at different stages of embryo development. Insulation scores are reported for boundaries found at 25 hpf. Insulation is negative at the strong TAD boundaries (fewer average interactions). Compartment scores are reported according to the 25 hpf reference annotation. P-values of one-sided Mann-Whitney unpaired test (*<0.1, **<0.01, see Source Data for precise values). Boxes represent the quartiles of the values; the center line is the median; whiskers extend to 1.5 times the interquartile range; notches show a confidence interval around the median.

d. Scaling plots for different stages.
(top) Dependence of the contact probability Pc(s) on the genomic distance (s).
(middle and bottom) First derivative indicating the slope of Pc(s). The region between 3 Kb and 1 Mb is additionally magnified (bottom). Arrows indicate the approximate positioning of the scaling humps, visible as broad elevations of interactions. The short-range peak for 2.75 hpf is almost absent, while it becomes more discernible at 5.3 hpf and shifts toward larger genomic separations. The peak reaches 100 Kb (typical for other vertebrates [1]) at 25 hpf.

e. Fountain calling with *fontanka* algorithm.
(top) log2 observed/expected ratios of manually picked fountains at 5.3 hpf are averaged to obtain a fountain mask.
(second top) The fountain mask is compared against 0.4 Mb sliding window, yielding a fountain score for each 10-Kb genomic bin.
(second bottom) The peaks in the fountain score (gray triangles) are considered fountains if their noise score is less than threshold (bottom). The 1460 fountains found on 5.3 hpf contact map using this procedure are referred to as fountains.

f. Setting up the thresholds of fountain peak score and noise score for selecting fountains, see Methods "Fountain calling with *fontanka*".

g. Scatter plot of protractor tool [2] and *fontanka* fountain score.

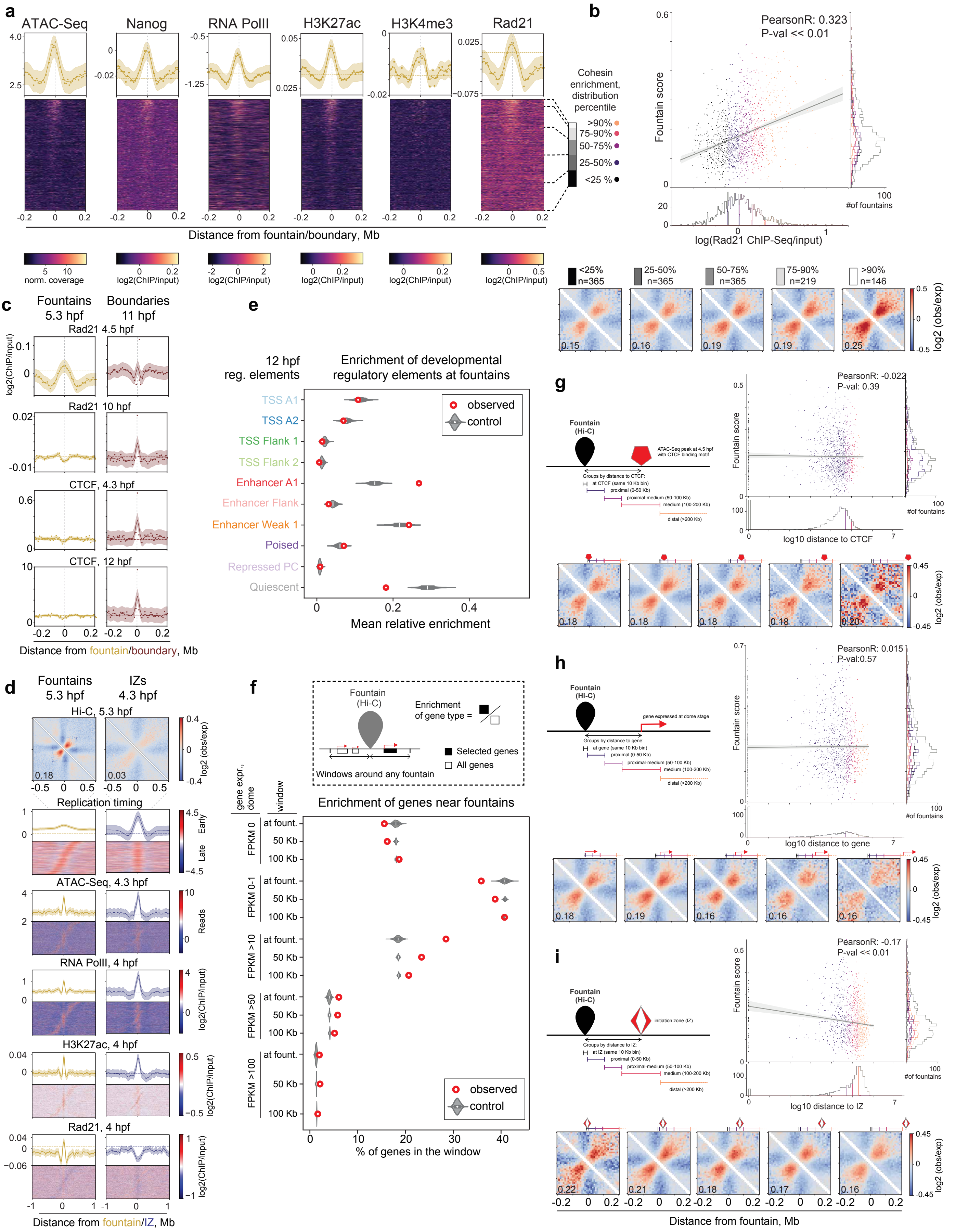

a
ATAC-Seq
Nanog
RNA PolII
H3K27ac
H3K4me3
Rad21
Distance from fountain/boundary, Mb
norm. coverage
log2(ChIP/input)
Cohesin enrichment, distribution percentile
>90%
75-90%
50-75%
25-50%
<25 %
b
PearsonR: 0.323
P-val << 0.01
Fountain score
#of fountains
log(Rad21 ChIP-Seq/input)
<25% n=365
25-50% n=365
50-75% n=365
75-90% n=219
>90% n=146
log2 (obs/exp)
c
Fountains 5.3 hpf
Boundaries 11 hpf
Rad21 4.5 hpf
Rad21 10 hpf
CTCF, 4.3 hpf
CTCF, 12 hpf
log2(ChIP/input)
Distance from fountain/boundary, Mb
d
Fountains 5.3 hpf
IZs 4.3 hpf
Hi-C, 5.3 hpf
Replication timing
Early
Late
ATAC-Seq, 4.3 hpf
Reads
RNA PolII, 4 hpf
H3K27ac, 4 hpf
Rad21, 4 hpf
Distance from fountain/IZ, Mb
e
12 hpf reg. elements
Enrichment of developmental regulatory elements at fountains
TSS A1
TSS A2
TSS Flank 1
TSS Flank 2
Enhancer A1
Enhancer Flank
Enhancer Weak 1
Poised
Repressed PC
Quiescent
observed
control
Mean relative enrichment
f
Fountain (Hi-C)
Enrichment of gene type =
Selected genes
All genes
Windows around any fountain
gene expr., dome
window
Enrichment of genes near fountains
FPKM 0
FPKM 0-1
FPKM >10
FPKM >50
FPKM >100
at fount.
50 Kb
100 Kb
% of genes in the window
g
Fountain (Hi-C)
ATAC-Seq peak at 4.5 hpf with CTCF binding motif
Groups by distance to CTCF:
at CTCF (same 10 Kb bin)
proximal (0-50 Kb)
proximal-medium (50-100 Kb)
medium (100-200 Kb)
distal (>200 Kb)
PearsonR: -0.022
P-val: 0.39
log10 distance to CTCF
h
gene expressed at dome stage
Groups by distance to gene:
at gene (same 10 Kb bin)
PearsonR: 0.015
P-val:0.57
log10 distance to gene
i
initiation zone (IZ)
Groups by distance to IZ:
at IZ (same 10 Kb bin)
PearsonR: -0.17
P-val << 0.01
log10 distance to IZ
Distance from fountain, Mb

Supplementary Figure 2.

Properties of fountains (n=1460).

a. Fountains associate with Rad21, RNA polymerase II, Nanog, and H3K27ac, and ATAC-seq open regions. Heatmaps are sorted by decreasing fountain score. Datasets as in Fig. 2a.

b. Fountain score correlates with cohesin.
(top) 4.5 hpf Rad21[3] ChIP-seq versus fountain score, colored by Rad21 percentiles. Each dot represents a single fountain; gray line shows linear regression.
(bottom) Average 5.3 hpf Hi-C pileups for Rad21-stratified fountain groups.

c. Rad21 and CTCF signal around fountains before and after gastrulation. ChIP-seq data for cohesin at 4.5 and 10 hpf from [3]. CTCF sites were inferred from ATAC-seq peaks containing CTCF motifs at 4.3 and 12 hpf [4] (Supplementary Methods "CTCF binding inference from ATAC-seq").

d. (related to Fig. 2f) Fountains are distinct from replication initiation zones (IZs). Profiles are shown around fountain bases (n=1460) and IZs at 4.3 hpf (from [5]). Only the nearest IZ per fountain base was used (n=1382). Fountains were sorted by distance to the nearest IZ and vice versa. Both fountains and IZs are enriched in enhancers, but only fountains are enriched in cohesin.

e. (related to Fig. 2c) Fountains colocalize with enhancers at 12 hpf (from [6]).

f. Fountains are preferentially enriched near actively transcribed zygotic genes. For each window size, we computed fraction of genes in each expression category within windows centered on fountains ("at fountain"; red points) and compared to a randomized gene categories control. Genes with expression levels from EBI Expression Atlas[7]; maternal transcripts (from [8]) were excluded.

(g-i). Fountain strength is independent of distance to inferred CTCF sites (4.3 hpf, g), TSSs of expressed genes (from [7], h), and IZs (4.3 hpf, from [5], i). For each feature: (top left) schematic defining the fountain groups by their distance to the nearest feature; (top right) scatter plot of fountain score versus distance to the nearest feature, with each point representing a fountain and gray line showing linear fit across all fountains; (bottom row) mean pileups for fountain groups by distance.

Source data are provided as a Source Data file.

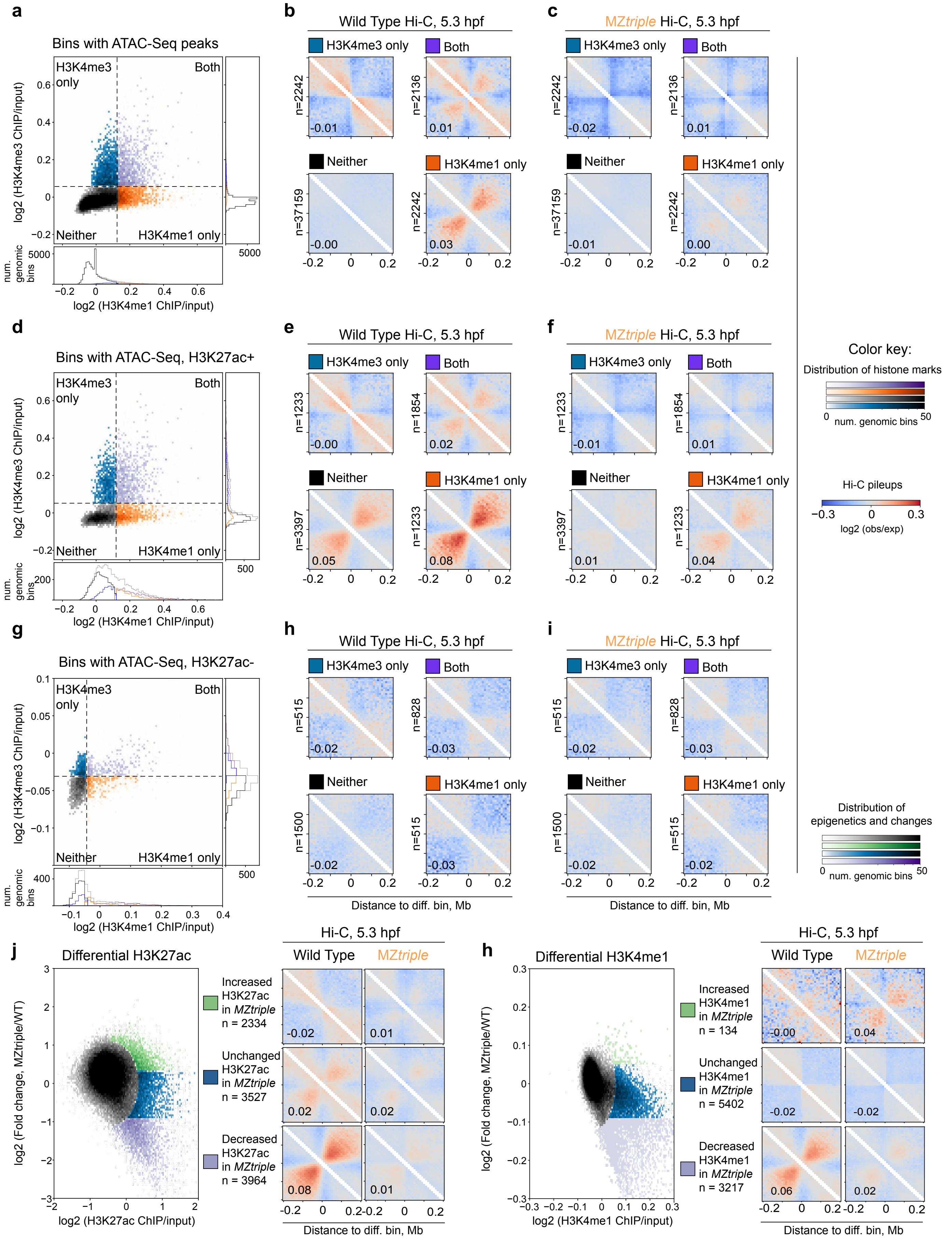

a
Bins with ATAC-Seq peaks
H3K4me3 only
Both
Neither
H3K4me1 only
log2 (H3K4me3 ChIP/input)
log2 (H3K4me1 ChIP/input)
num. genomic bins
b
Wild Type Hi-C, 5.3 hpf
c
MZtriple Hi-C, 5.3 hpf
d
Bins with ATAC-Seq, H3K27ac+
e
Wild Type Hi-C, 5.3 hpf
f
MZtriple Hi-C, 5.3 hpf
g
Bins with ATAC-Seq, H3K27ac-
h
Wild Type Hi-C, 5.3 hpf
i
MZtriple Hi-C, 5.3 hpf
Distance to diff. bin, Mb
Color key:
Distribution of histone marks
num. genomic bins
Hi-C pileups
log2 (obs/exp)
Distribution of epigenetics and changes
j
Differential H3K27ac
log2 (Fold change, MZtriple/WT)
log2 (H3K27ac ChIP/input)
Hi-C, 5.3 hpf
Wild Type
MZtriple
Increased H3K27ac in MZtriple n = 2334
Unchanged H3K27ac in MZtriple n = 3527
Decreased H3K27ac in MZtriple n = 3964
h
Differential H3K4me1
log2 (H3K4me1 ChIP/input)
Increased H3K4me1 in MZtriple n = 134
Unchanged H3K4me1 in MZtriple n = 5402
Decreased H3K4me1 in MZtriple n = 3217

Supplementary Figure 3.

Fountains form at accessible chromatin enriched in H3K4me1 and H3K27ac and depend on pioneer transcription factors Pou5f3, Sox19b, and Nanog.

The mean fountain score is shown in the top-right corner of each Hi-C pileup.

(a-i). Accessible regions in wild-type embryos at 4.3 hpf (from [9]) were sorted by enrichment in H3K4me1, H3K4me3, and H3K27ac at 4 hpf [10]. Related to Figs. 2d and 3b.

Rows: (a-c) All ATAC-seq peaks, n=43779; (d-f) H3K27ac-high peaks (top 10% by H3K27ac among all peaks), n=7717; (g-i) H3K27ac-low peaks (bottom 10% by H3K27ac among all peaks), n=3358.

Columns: (a,d,g). Scatterplots and four groups ("Neither", "Both", "H3K4me1 only", "H3K4me3 only"), defined by the 90th-percentile thresholds on each axis. (b,e,h). Mean on-diagonal pileups for four groups in 5.3 hpf wild type. In wild type, fountains are detected in the "H3K4me1 only" and "Both" groups, with the strongest signal in H3K27ac-high "H3K4me1 only" regions (active enhancers). H3K27ac-low accessible regions lack fountains. (c,f,i) Mean pileups for four groups in 5.3 hpf MZ*triple*. Fountain is absent/severely reduced in all groups.

(j-k). Changes in H3K27ac (j) and H3K4me1 (i) in MZ*triple* mutant are coupled with fountain formation. Related to Figs. 2c,d.

Left: distributions and bin groups by response; right: average Hi-C pileups for each group in wild type and MZ*triple* at 5.3 hpf.

Source data are provided as a Source Data file.

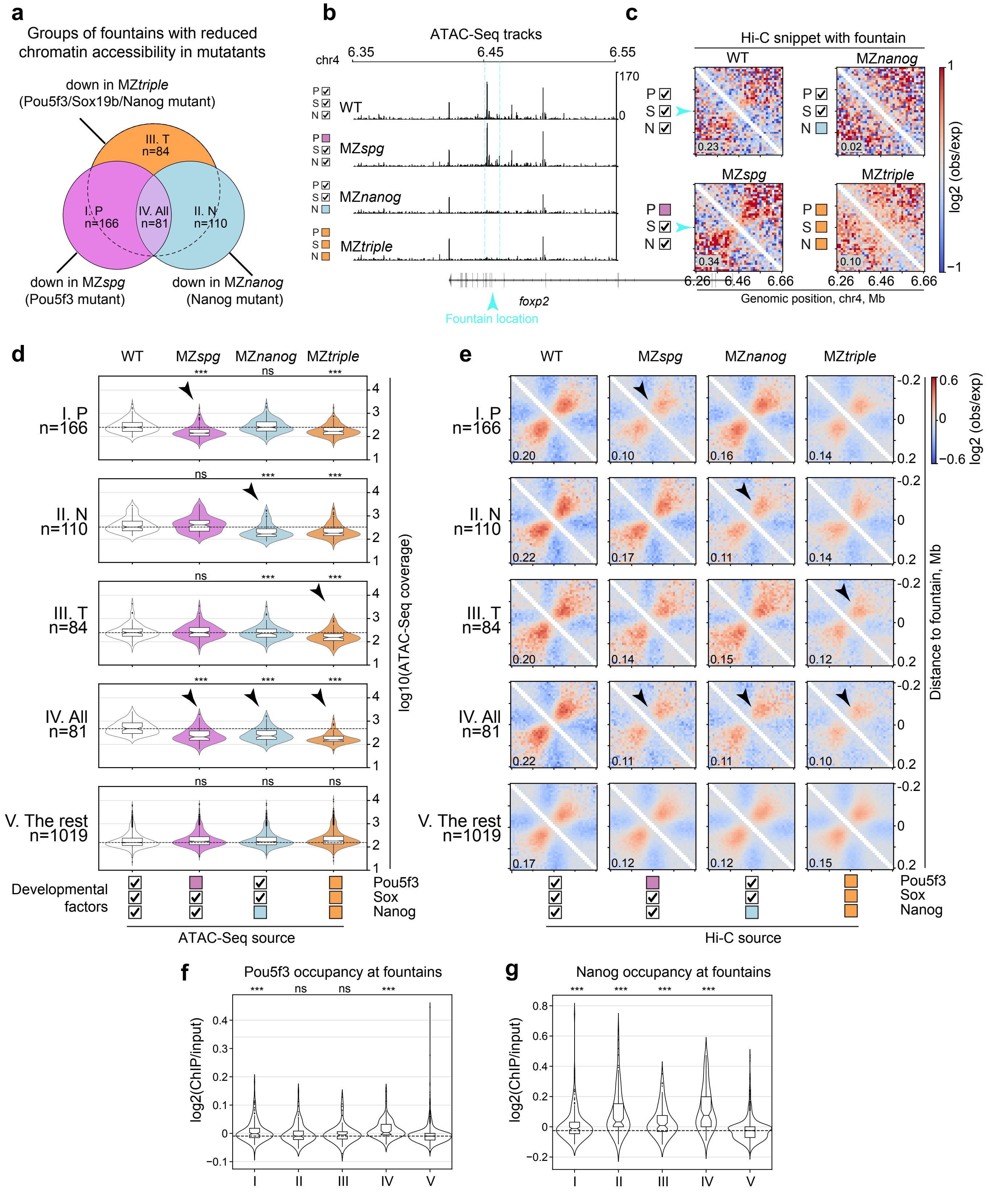
a
Groups of fountains with reduced chromatin accessibility in mutatants
down in MZtriple (Pou5f3/Sox19b/Nanog mutant)
III. T n=84
I. P n=166
IV. All n=81
II. N n=110
down in MZspg (Pou5f3 mutant)
down in MZnanog (Nanog mutant)
b
ATAC-Seq tracks
chr4
6.35
6.45
6.55
170
0
WT
MZspg
MZnanog
MZtriple
foxp2
Fountain location
c
Hi-C snippet with fountain
WT
MZnanog
MZspg
MZtriple
0.23
0.02
0.34
0.10
log2 (obs/exp)
6.26
6.46
6.66
Genomic position, chr4, Mb
d
WT
MZspg
MZnanog
MZtriple
I. P n=166
II. N n=110
III. T n=84
IV. All n=81
V. The rest n=1019
log10(ATAC-Seq coverage)
Developmental factors
Pou5f3
Sox
Nanog
ATAC-Seq source
e
WT
MZspg
MZnanog
MZtriple
log2 (obs/exp)
Distance to fountain, Mb
Hi-C source
f
Pou5f3 occupancy at fountains
log2(ChIP/input)
g
Nanog occupancy at fountains
log2(ChIP/input)

Supplementary Figure 4.

Pioneer activities of Pou5f3 and Nanog separately contribute to the fountain formation (related to Fig. 5).

Source data are provided as a Source Data file.

a. Venn diagram defining fountain groups (I–IV) based on whether chromatin accessibility at 10 Kb fountain bases requires Pou5f3, Nanog, or both. Groups were assigned by the differential analysis of normalized ATAC-seq reads per 10 Kb fountain base using 4.3 hpf ATAC-seq from wild-type and mutants (from [8]).

(b, c). Example fountain with both chromatin accessibility (b) and fountain structure (c) regulated by Nanog but not Pou5f3.

b. ATAC-seq (normalized reads) in a 200 Kb window around the 10 Kb fountain base (blue arrows/dotted lines) in wild type, MZ*nanog*, MZ*spg*, and MZ*triple* (4.3 hpf; from [8]). Accessibility is lost only in MZ*nanog* and MZ*triple*.

c. Hi-C in a 400 Kb window centered on the fountain: the structure is present in wild type and MZ*spg* but lost in MZ*nanog* and MZ*triple*, accompanying the changes in accessibility.

d. Violin plots of ATAC-seq (4.3 hpf, from [8]) signal for five groups: I–IV as in (a); V (the rest) comprises fountains not requiring Pou5f3/Nanog for chromatin accessibility. Dashed lines indicate the wild-type median; arrowheads mark the genotypes used for group assignment. P-values of one-sided paired Wilcoxon signed-rank test (ns – not significant, ***<0.001). Group definitions and signals are provided in Supplementary Dataset 5, precise p-values provided in Source Data).

e. Average on-diagonal Hi-C pileups for groups in (d), with fountain scores, demonstrating matched regulation of accessibility (black arrowheads) and fountain strength.

(f, g). Pou5f3 and Nanog are enriched at fountains that they regulate. ChIP-seq signals for Pou5f3 (f) and Nanog (g) at 10 Kb fountain bases for the groups I-V. Each TF is enriched the most at the fountain group that it regulates: Pou5f3 at I.P and III.T groups, Nanog at II.N and III.T groups. Dashed lines show the median occupancy for non-regulated group V (lowest in both cases). P-values of one-sided Mann-Whitney unpaired test (ns – not significant, ***<0.001, precise values provided in Source Data); p-value in 1-way ANOVA < 2e-16 in both cases.

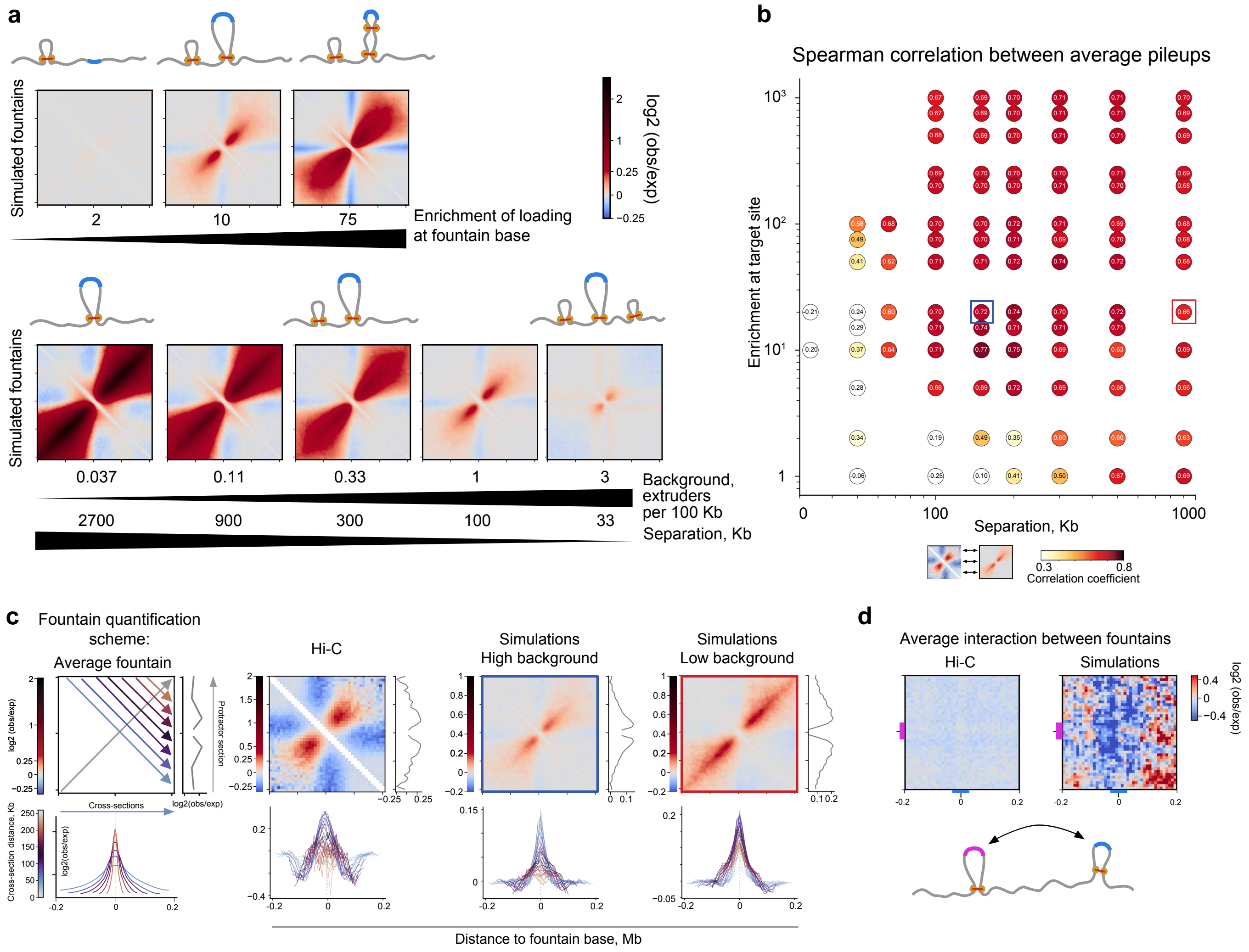
a
Simulated fountains
2
10
75
Enrichment of loading at fountain base
log2 (obs/exp)
Simulated fountains
0.037
0.11
0.33
1
3
Background, extruders per 100 Kb
2700
900
300
100
33
Separation, Kb
b
Spearman correlation between average pileups
Enrichment at target site
Separation, Kb
Correlation coefficient
c
Fountain quantification scheme:
Average fountain
Cross-sections
Protractor section
log2(obs/exp)
Cross-section distance, Kb
Hi-C
Simulations
High background
Simulations
Low background
Distance to fountain base, Mb
d
Average interaction between fountains
Hi-C
Simulations
log2 (obs/exp)

Supplementary Figure 5.

Simulations of facilitated cohesin loading (related to Fig. 5).

(a-b). Parameter sweep for the model of facilitated extruder loading with background extruders.

a. Average fountains for simulations with different parameters of facilitated extrusion.

b. Heatmap of Spearman correlation coefficient between Hi-C and simulated average fountains (detailed heatmap for Fig. 5e). Boxes represent the set of parameters selected for figure (c), blue – best parameters resulting in dispersive fountain, red – worse parameters resulting in non-dispersive fountain.

c. Fountain quantification for real Hi-C data and two simulations from (b). (left) Scheme of quantification: calculation of protractor (right subplot) and cross-sections (bottom subplot) of the average fountain. Experimental Hi-C data (second from left) resembles disperse fountains formed in the presence of the background loading (third from left) but is less similar to the non-disperse model of fountains (right). The difference between models can be explained as the growth of dispersion of Hi-C signal enrichment with genomic distance, noticeable at cross-sections (bottom subplots). Note how both Hi-C and simulated dispersive fountains' cross-sections are similar in terms of broadening with distance. The parameters for simulations are as in (b).

d. Fountain-fountain interactions in Hi-C data (left) and simulations (right). Absence of enriched interactions between fountains rules out a compartment-like mechanism of fountain formation, where fountains could be formed by affinities of fountain-proximal regions.

In the potential compartment-like mechanism, which we do not explore in the current work, two genomic regions (magenta and blue) have affinity for each other and form fountains around them. However, these “sticky” regions of different fountain bases would also generate enrichment of interactions between fountains. We examined Hi-C contacts between fountains in real Hi-C data (left) and found no such enrichment, ruling out the affinity-mediated mechanism. The same absence of fountain-fountain interactions can be detected in the simulations of facilitated extrusion (right).

Source data are provided as a Source Data file.

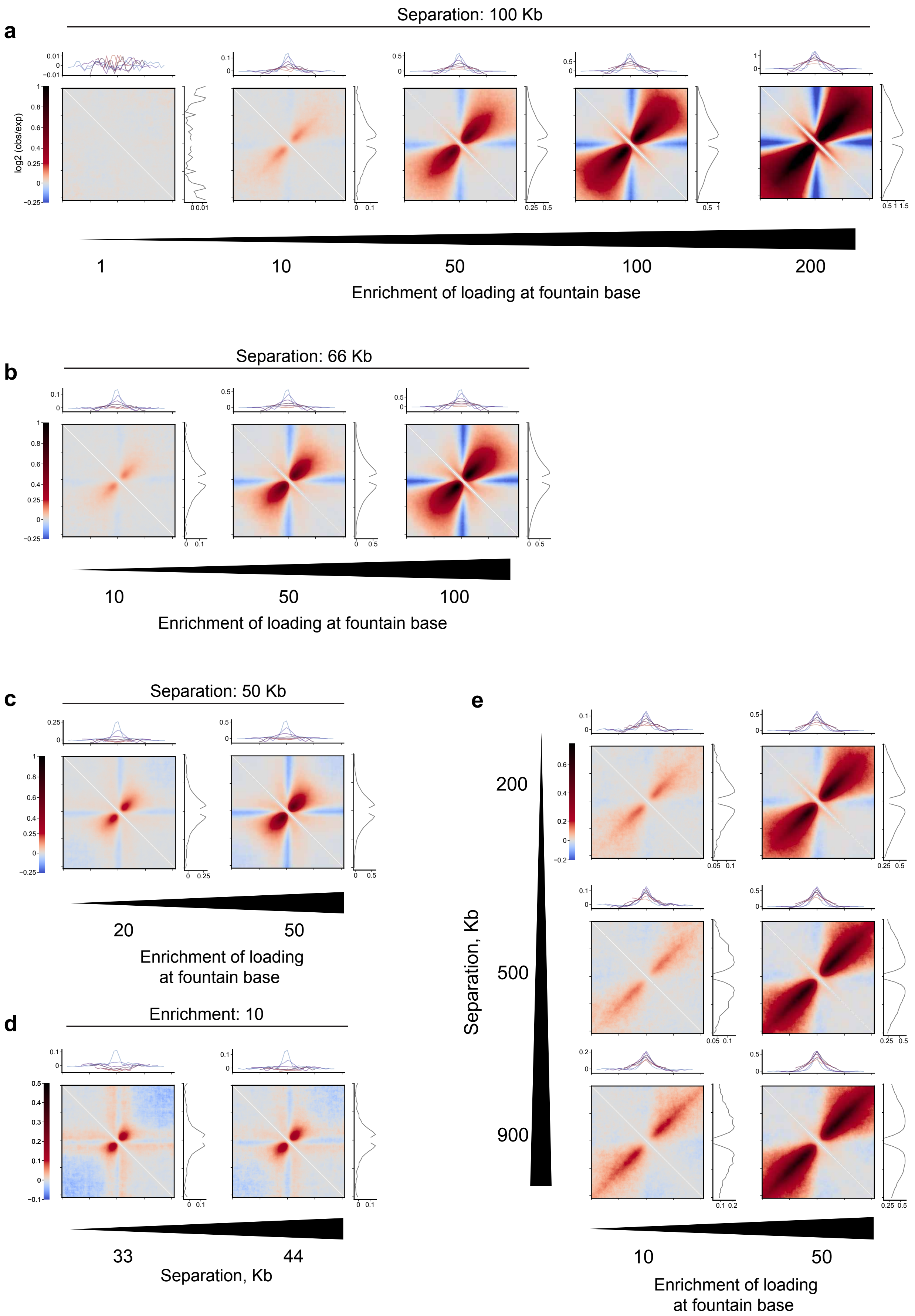

a
Separation: 100 Kb
log2 (obs/exp)
1
10
50
100
200
Enrichment of loading at fountain base
b
Separation: 66 Kb
10
50
100
Enrichment of loading at fountain base
c
Separation: 50 Kb
20
50
Enrichment of loading
at fountain base
d
Enrichment: 10
33
44
Separation, Kb
e
Separation, Kb
200
500
900
10
50
Enrichment of loading
at fountain base

Supplementary Figure 6.

Fountain shapes in different modes of polymer simulations of fountains for the model of facilitated extruder loading with background extruders (Supporting material for the main Fig. 5).

Each plot element:
(top) Cross-sections of the simulated fountain as defined in Supplementary Fig. 5c.
(center) Simulated average fountain.
(right) Protractor of the simulated fountain as defined in Supplementary Fig. 5c.

a. Simulated fountains for various enrichments at the fountain base with fixed separation of 100 Kb, extended Supplementary Fig. 5a.

b. Simulated fountains for various enrichments at the fountain base with fixed separation of 66 Kb. Note that here, the fountain shape changes slightly differently than in (a), which can be attributed to more cohesins in the background than in (a).

c. Simulated fountains for various enrichments at the fountain base with fixed separation of 50 Kb. Note that here, the fountain shape changes slightly differently than in (a,b), which can be attributed to more cohesins in the background than in (a,b); the fountain is more suppressed.

d. Simulated fountains for two small separations of background extruders with fixed enrichment at the fountain base of 10, extended Supplementary Fig. 5a. Note how the fountain is generally suppressed. Two stripes emerge from the base at smaller separations (33 Kb) due to a large number of stalled background extruders.

c. Simulated fountains for varying both enrichment and separation.

Source data are provided as a Source Data file.

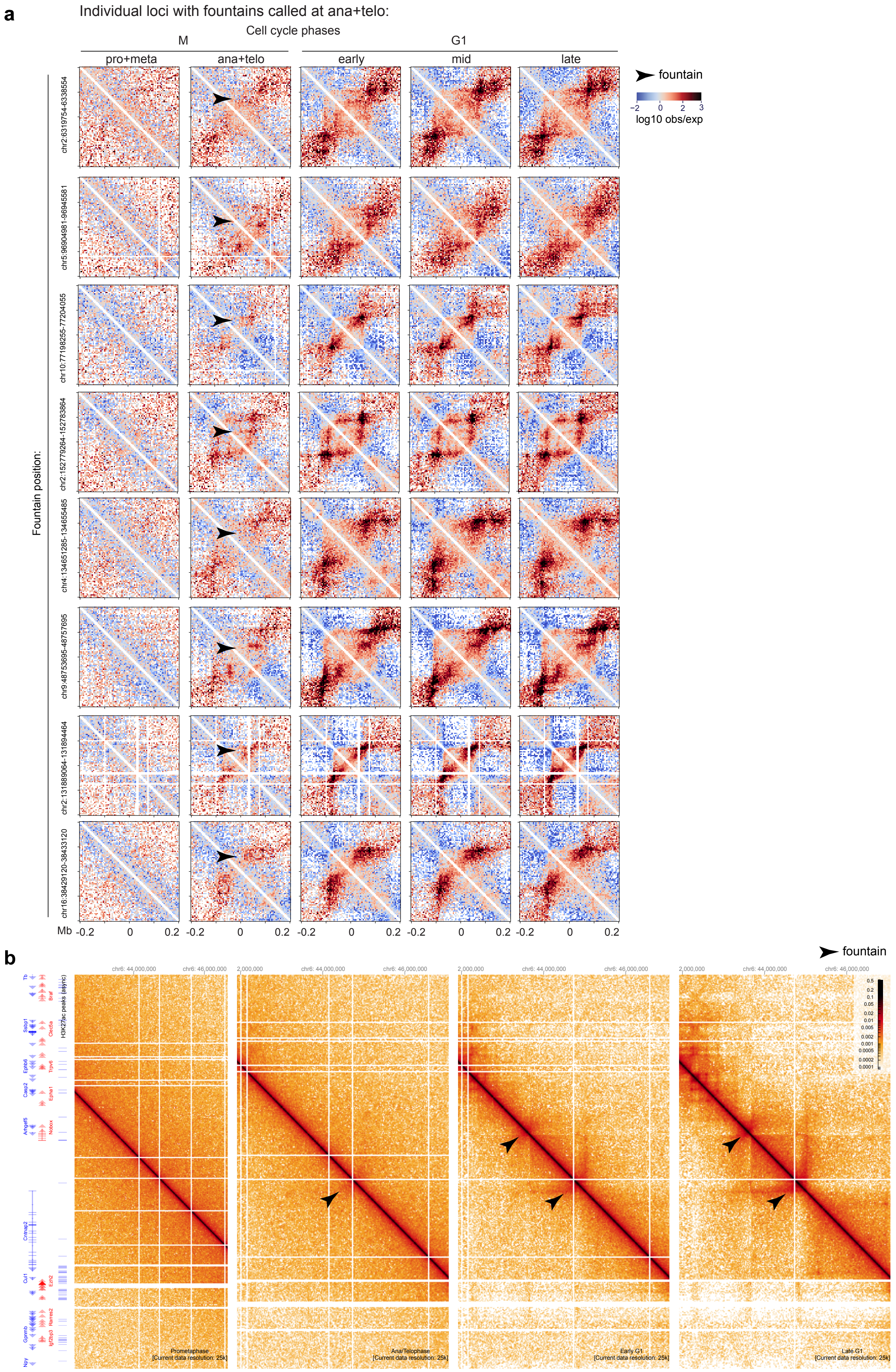
a
Individual loci with fountains called at ana+telo:
Cell cycle phases
M
G1
pro+meta
ana+telo
early
mid
late
fountain
log10 obs/exp
−2
0
2
3
Fountain position:
chr2:6319754-6338554
chr5:96904981-96945581
chr10:77198255-77204055
chr2:152779264-152783864
chr4:134651285-134655485
chr9:48753695-48757695
chr2:131889064-131894464
chr16:38429120-38433120
Mb
-0.2
0
0.2
b
fountain
chr6: 44,000,000
chr6: 46,000,000
2,000,000
H3K27ac peaks (async)
Prometaphase
[Current data resolution: 25k]
Ana/Telophase
[Current data resolution: 25k]
Early G1
[Current data resolution: 25k]
Late G1
[Current data resolution: 25k]

Supplementary Figure 7.

Examples of individual fountains in G1E erythroblast cell line emerging in the cell cycle.

a. Top fountains called based on H3K27ac peaks with prominent fountain score peak. Hi-C maps are shown in logarithm observed-over-expected units.

b. HiGlass [11] genome browser view on the genomic region with two fountains in mouse G1E erythroblast cell line emerging in the cell cycle. (Central) Hi-C for four stages of cell cycle from [12] with (left) gene track and H3K27ac peaks from [13]. Hi-C maps are shown in Hi-C contacts normalized by iterative correction.

Mouse genome coordinates (mm10) are shown above the maps. Hi-C bin size is 25Kb. Arrows mark the locations of two fountains emerging at ana/telophase and early G1 and later transforming into other chromatin structures (TAD and stripe, correspondingly).

Source data are provided as a Source Data file.

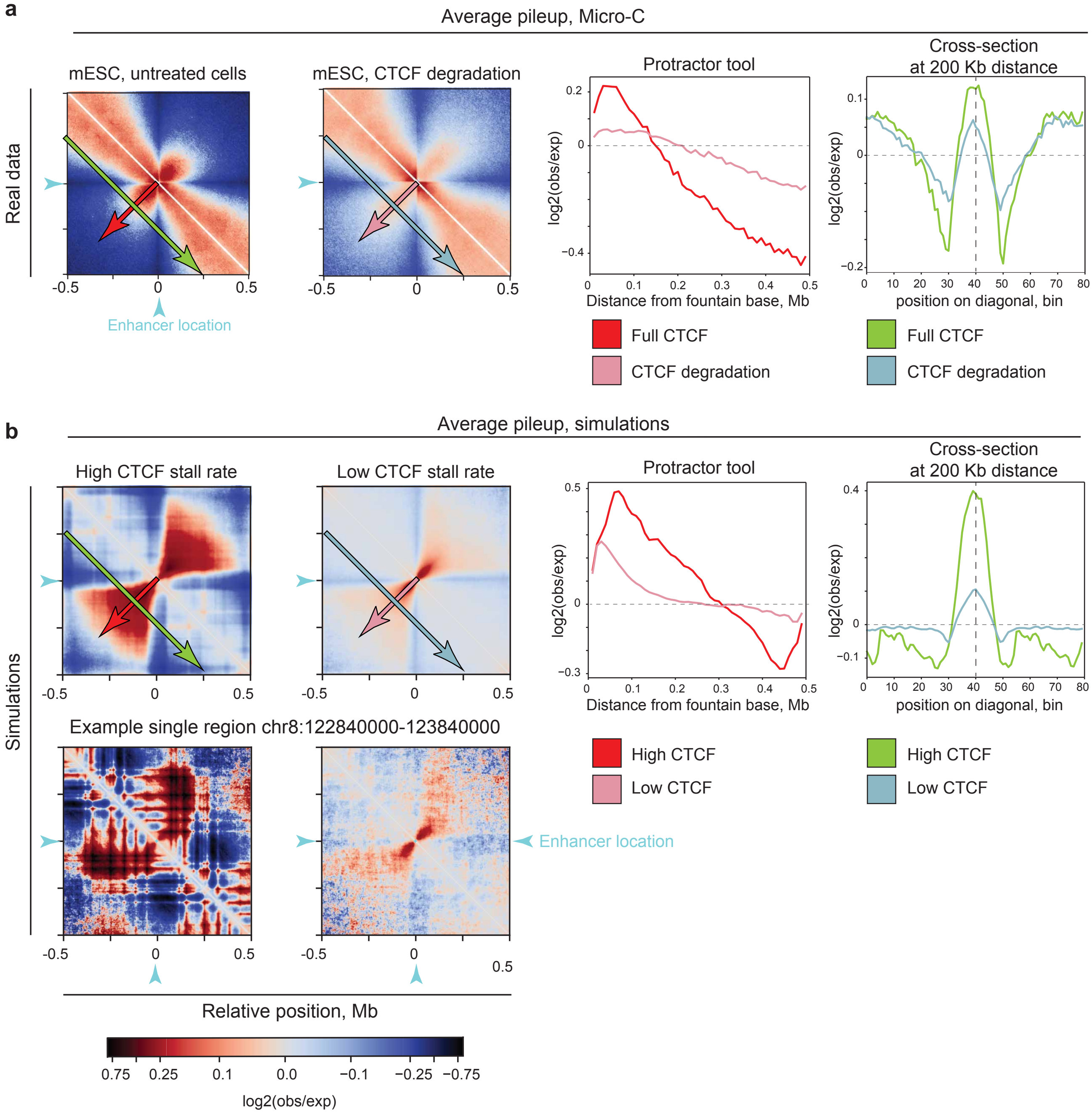

a
Average pileup, Micro-C
mESC, untreated cells
mESC, CTCF degradation
Real data
Enhancer location
Protractor tool
log2(obs/exp)
Distance from fountain base, Mb
Cross-section
at 200 Kb distance
position on diagonal, bin
Full CTCF
CTCF degradation
b
Average pileup, simulations
High CTCF stall rate
Low CTCF stall rate
Simulations
Example single region chr8:122840000-123840000
Enhancer location
High CTCF
Low CTCF
Relative position, Mb
0.75 0.25 0.1 0.0 −0.1 −0.25 −0.75
log2(obs/exp)

Supplementary Figure 8.

Fountain signatures with and without CTCF, analyzed in comparison with experimental mESC Micro-C data [15] and realistic simulations of the mouse genome. Realistic simulations were performed with the same parameters as the best hit for zebrafish simulations. In these simulations, we placed facilitated loading sites at mESC enhancers (ENCODE data [16]) and included oriented CTCF peaks (from [15]) as extrusion barriers (CTCF orientation inferred by motif calling, as in Fig. 6a).

a. Experimental mESC Micro-C. Average enhancer pileups are shown for untreated cells and for the CTCF-degradation condition, together with the corresponding protractor metric and a cross-section measured at 200 Kb. Arrows indicate the protractor and cross-section tools on the heatmaps.
(left) Average pileup at enhancers in untreated cells.
(second left) Average pileup at enhancers in the CTCF degradation dataset.
(second right) Protractor of average pileup.
(right) Cross-section of average pileup at 200 Kb distance of average pileup.

b. Simulations. Fountain signature in simulated data. Arrows in the heatmaps show the protractor and cross-section tools.

Top row: average pileup for multiple simulated regions. With strong CTCF stalling (stall probability 0.25), extruders stall at CTCF and produce a grid-like pattern of dots that overlays the fountain. With weak stalling (0.01), extrusion is minimally impeded, altering the fountain signature; the protractor reveals a more extended but weaker signal, and the 200 Kb cross-section shows reduced central-peak intensity.
(left) Average simulated pileup at enhancers, high CTCF stall rate (0.25, n=366 simulated regions).
(second left) Average simulated pileup at enhancers, low CTCF stall rate (0.01, n=350 simulated regions).
(second right) Protractor of average pileup.
(right) Cross-section of average pileup at 200 Kb distance of average pileup.

Bottom row: an example simulated region from chromosome 8, high CTCF stall rate (0.25, left) and low CTCF stall rate (0.01, right). Note how the fountain becomes more noticeable when there are no CTCF-dependent dots.

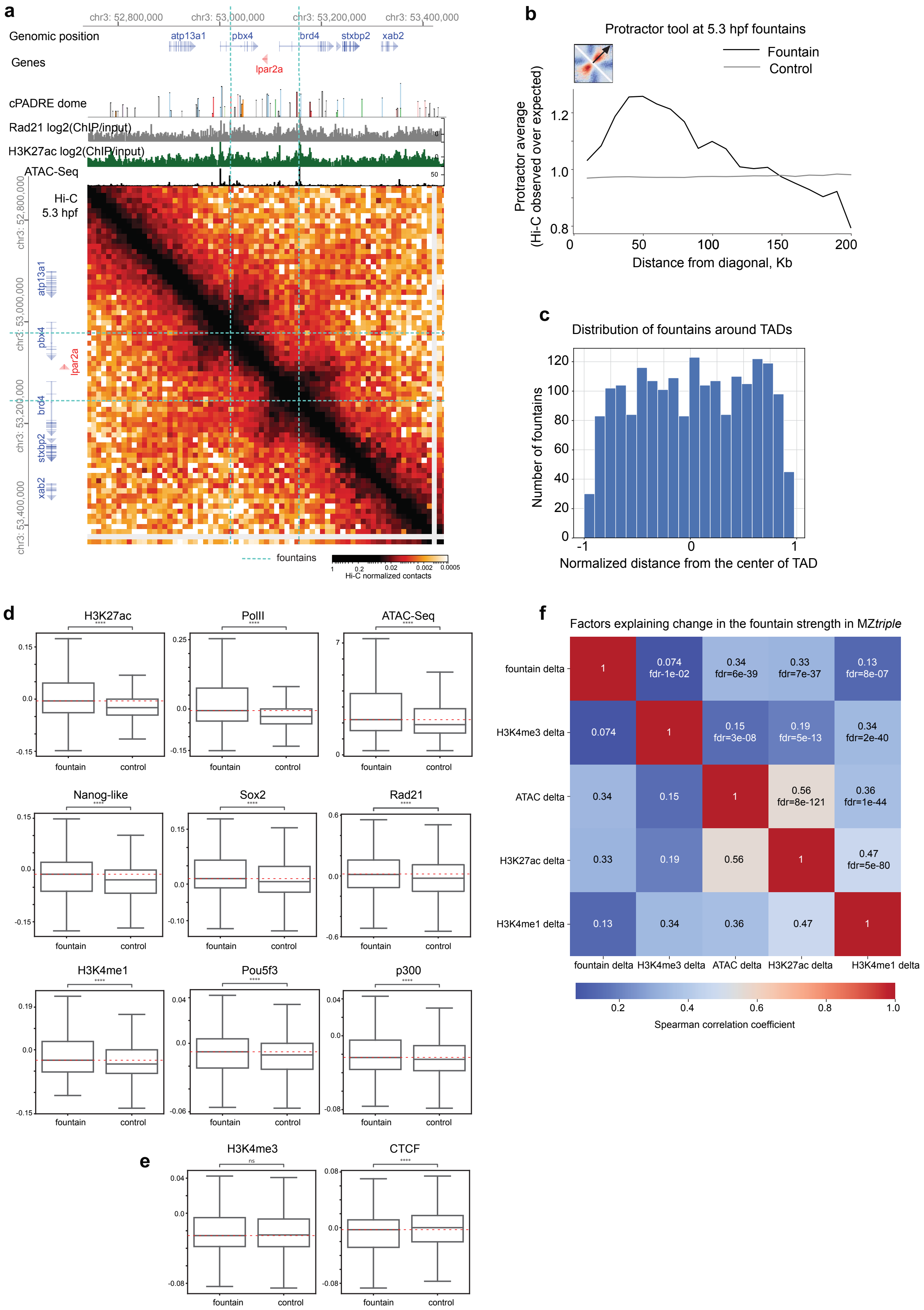
a
chr3: 52,800,000
chr3: 53,000,000
chr3: 53,200,000
chr3: 53,400,000
Genomic position
Genes
atp13a1
pbx4
brd4
stxbp2
xab2
lpar2a
cPADRE dome
Rad21 log2(ChIP/input)
H3K27ac log2(ChIP/input)
ATAC-Seq
50
Hi-C
5.3 hpf
fountains
Hi-C normalized contacts
b
Protractor tool at 5.3 hpf fountains
Fountain
Control
Protractor average
(Hi-C observed over expected)
Distance from diagonal, Kb
c
Distribution of fountains around TADs
Number of fountains
Normalized distance from the center of TAD
d
H3K27ac
PolII
ATAC-Seq
Nanog-like
Sox2
Rad21
H3K4me1
Pou5f3
p300
fountain
control
e
H3K4me3
CTCF
ns
f
Factors explaining change in the fountain strength in MZ*triple*
fountain delta
H3K4me3 delta
ATAC delta
H3K27ac delta
H3K4me1 delta
0.074
fdr-1e-02
0.34
fdr=6e-39
0.33
fdr=7e-37
0.13
fdr=8e-07
0.15
fdr=3e-08
0.19
fdr=5e-13
fdr=2e-40
0.56
fdr=8e-121
0.36
fdr=1e-44
0.47
fdr=5e-80
Spearman correlation coefficient

Supplementary Figure 9.

Fountains validation and their properties.

a. HiGlass [11] view with two fountains at 5.3 hpf of zebrafish development. Shown: genes, annotation of developmental regulatory elements (ChromHMM at dome ATAC-Seq peaks from [6]), epigenetic annotations, Hi-C map (10 Kb resolution). Colors same as in Fig. 2c (TSS A1 and A2 – blue, TSS Flank 1 and 2 – green, Enhancer – red, Enhancer flank – rose, Enhancer Weak 1 – orange, Poised – purple, Repressed – light-purple, and Quiescent – gray).

b. Protractor tool [2] for validation of fountains. Inset: Hi-C snippet with a perpendicular to the main diagonal (protractor for ideal hairpin). The protractor shows the mean values in Hi-C snippets at fountains along the represented direction. Note a hump at 50 Kb (peak intensity of fountain in observed over expected units). Control: same for non-fountain genomic regions.

c. Distribution of fountains around centers of TADs show no correlation. To determine individual TADs, we paired sequential pairs of the boundaries at 11 hpf (confirmed by CTCF presence).

(d-f) Factors associated with fountains and fountain strength.

d. (Related to Fig. 2a) Boxplots of epigenetic signal at fountains versus control for different datasets from Fig. 2a, ordered by the significance of the difference in means (from upper left to bottom right). Control: bins with similar levels of chromatin openness excluding fountains +-10Kb offset. Only those characteristics that are positively associated with fountains are shown (p-value<0.05 for the Mann-Whitney test with a greater alternative after Benjamini-Yakuteli multiple testing correction, *** – p-value<0.001, ns – not significant). Red dashed line: observed median for fountains. Boxes represent the quartiles of the values; the center line is the median; whiskers extend to 1.5 interquartile range.

e. Same boxplots for H3K4me1, which is not significantly associated with fountains, and CTCF, which is negatively associated with fountains.

f. (Related to Fig. 3e) Spearman correlation of epigenetic changes with fountain score change in MZ*triple* mutant. FDR-adjusted p-values (Benjamini-Yakuteli) computed from Beta-test p-values (Beta test via *scipy* [14]).

Source data are provided as a Source Data file.

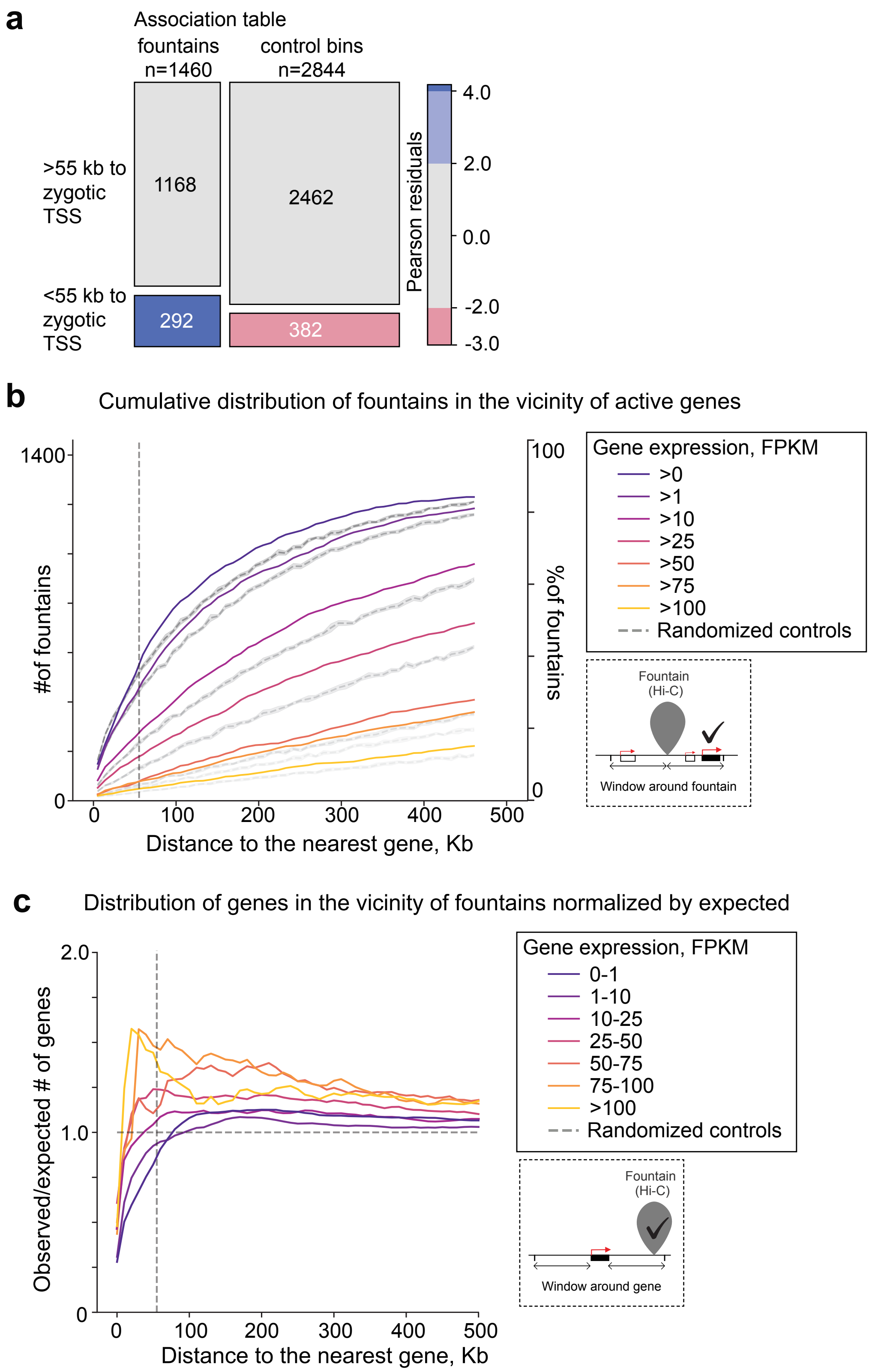
a
Association table
fountains
n=1460
control bins
n=2844
>55 kb to zygotic TSS
<55 kb to zygotic TSS
1168
2462
292
382
Pearson residuals
4.0
2.0
0.0
-2.0
-3.0
b
Cumulative distribution of fountains in the vicinity of active genes
1400
0
#of fountains
100
0
%of fountains
0
100
200
300
400
500
Distance to the nearest gene, Kb
Gene expression, FPKM
>0
>1
>10
>25
>50
>75
>100
Randomized controls
Fountain
(Hi-C)
Window around fountain
c
Distribution of genes in the vicinity of fountains normalized by expected
2.0
1.0
0
Observed/expected # of genes
0
100
200
300
400
500
Distance to the nearest gene, Kb
Gene expression, FPKM
0-1
1-10
10-25
25-50
50-75
75-100
>100
Randomized controls
Fountain
(Hi-C)
Window around gene

Supplementary Figure 10.

Association of fountains with zygotic genes, related to Suppementary. Figures 2f, h.

a. Fountains preferentially localize near zygotic genes. Association table between fountains/control bins and transcription start sites (TSS) of zebrafish. Control: 10 Kb bins at the distances +/- 1 Mb from 1460 fountains, with excluded overlaps with fountains. The number of zygotic genes in the embryo (n=4777) and RNA-seq data are from [8]. Gene association rule: bin overlaps +/- 55 Kb from TSS. Number of cases in the table: 4304. Number of factors: 2. Chi-squared test for independence of all factors: chi-squared value 32, degrees of freedom 1, p-value 1.4e-08.

(b-c) Fountains preferentially localize near active zygotic genes and vice versa: active genes localize near fountains. More actively transcribed genes are enriched in fountains at distances below 100 Kb near them. Genes with little or no expression tend to have fewer fountains around them at distances below 50-100 Kb than expected at random.

b. Cumulative distribution of fountains in the vicinity of active genes. Gene expression is from EBI expression atlas [7]. Potential maternal transcripts (from [8]) were excluded. Control: randomly picked genomic regions, average for n=100 rounds. Vertical line: 55 Kb distance.

c. Distribution of genes near fountains normalized by expected at random (n=100 sampling rounds). Horizontal line: line of no enrichment. Above the horizontal line: enrichment over expected; below the horizontal line: depleted. Vertical line: 55 Kb distance.

Source data are provided as a Source Data file.

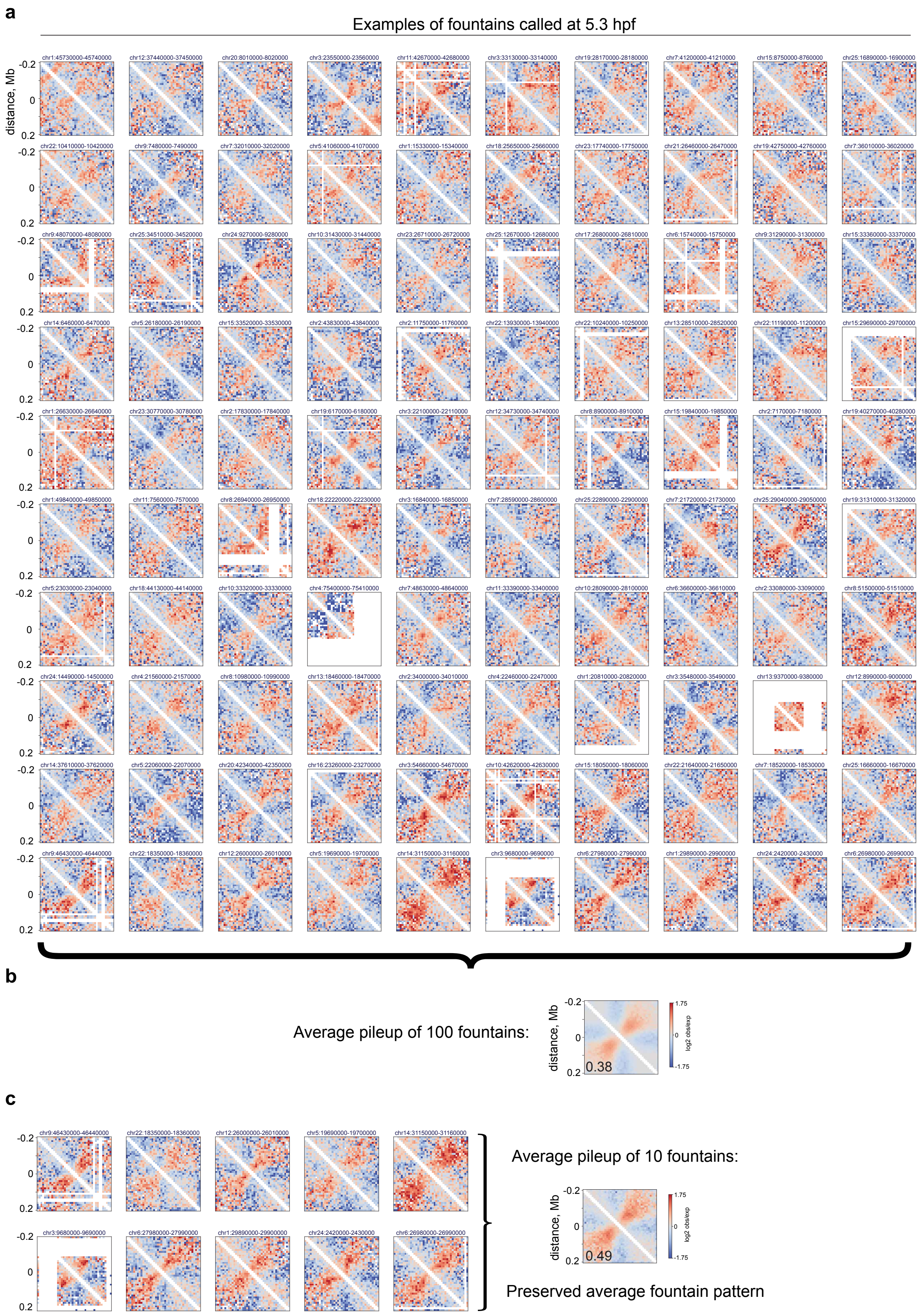
a
Examples of fountains called at 5.3 hpf
distance, Mb
-0.2
0
0.2
b
Average pileup of 100 fountains:
distance, Mb
0.38
1.75
0
-1.75
log2 obs/exp
c
Average pileup of 10 fountains:
distance, Mb
0.49
1.75
0
-1.75
log2 obs/exp
Preserved average fountain pattern

Supplementary Figure 11.

Averaging of multiple fountains result in average fountain pattern even upon sampling.
(a-b) Averaging 100 individual fountains at 5.3 hpf.
a. Snippets of Hi-C maps of n=100 fountain bases called at 5.3 hpf. Each snippet is named by the genomic position of the fountain base.
b. Average pileup of n=100 Hi-C snippets with average fountain. The average resembles individual conformations. The number in the corner represents the strength of the fountain signature. Number represents fountain strength of the average pileup.
c. Averaging n=10 snippets of 5.3 hpf Hi-C maps with fountain bases called at 5.3 hpf. Averaging results in average fountain.

Source data are provided as a Source Data file.

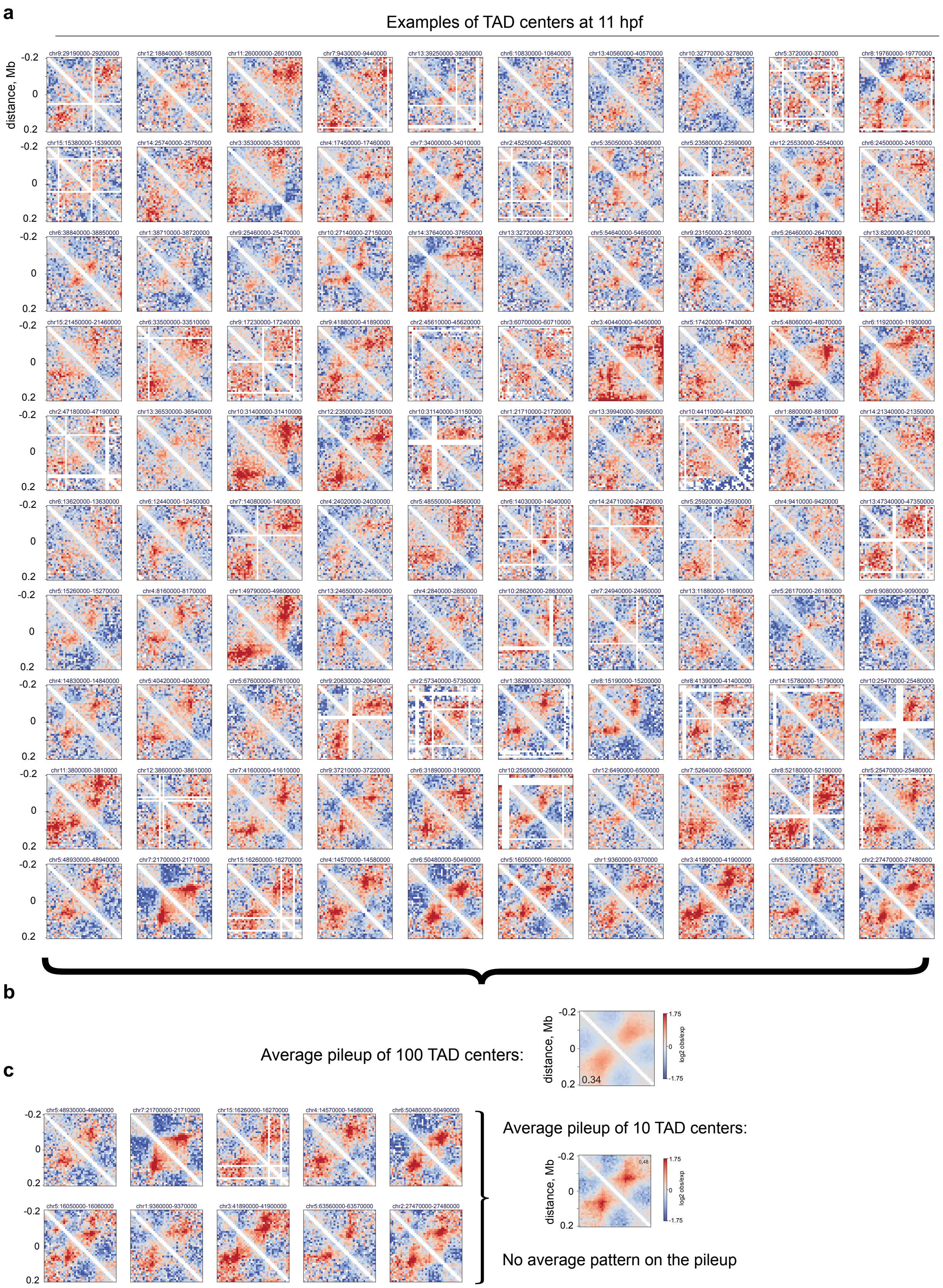
a
Examples of TAD centers at 11 hpf
distance, Mb
-0.2
0
0.2
b
Average pileup of 100 TAD centers:
0.34
1.75
-1.75
log2 obs/exp
c
Average pileup of 10 TAD centers:
0.48
No average pattern on the pileup

Supplementary Figure 12.

Averaging of multiple TAD centers result in average fountain pattern as well, but strategies like sampling and visual inspection can help spot the issue.
(a-b) Averaging 100 centers of TADs at 11 hpf.
a. Snippets of Hi-C maps of n=100 centers of TADs called at 11 hpf. Each snippet is named by the genomic position of the TAD center.
b. Average pileup of n=100 centers of TADs with fountain-like-looking output. The average does not resemble individual conformations. Number represents fountain strength of the average pileup.
c. Snippets of 11 hpf Hi-C maps of n=10 centers of TADs called at 11 hpf, all TADs have similar sizes. Averaging results in average TAD with a strong corner dot.

Source data are provided as a Source Data file.

Examples of fountains called at stage 11 *Xenopus tropicalis*

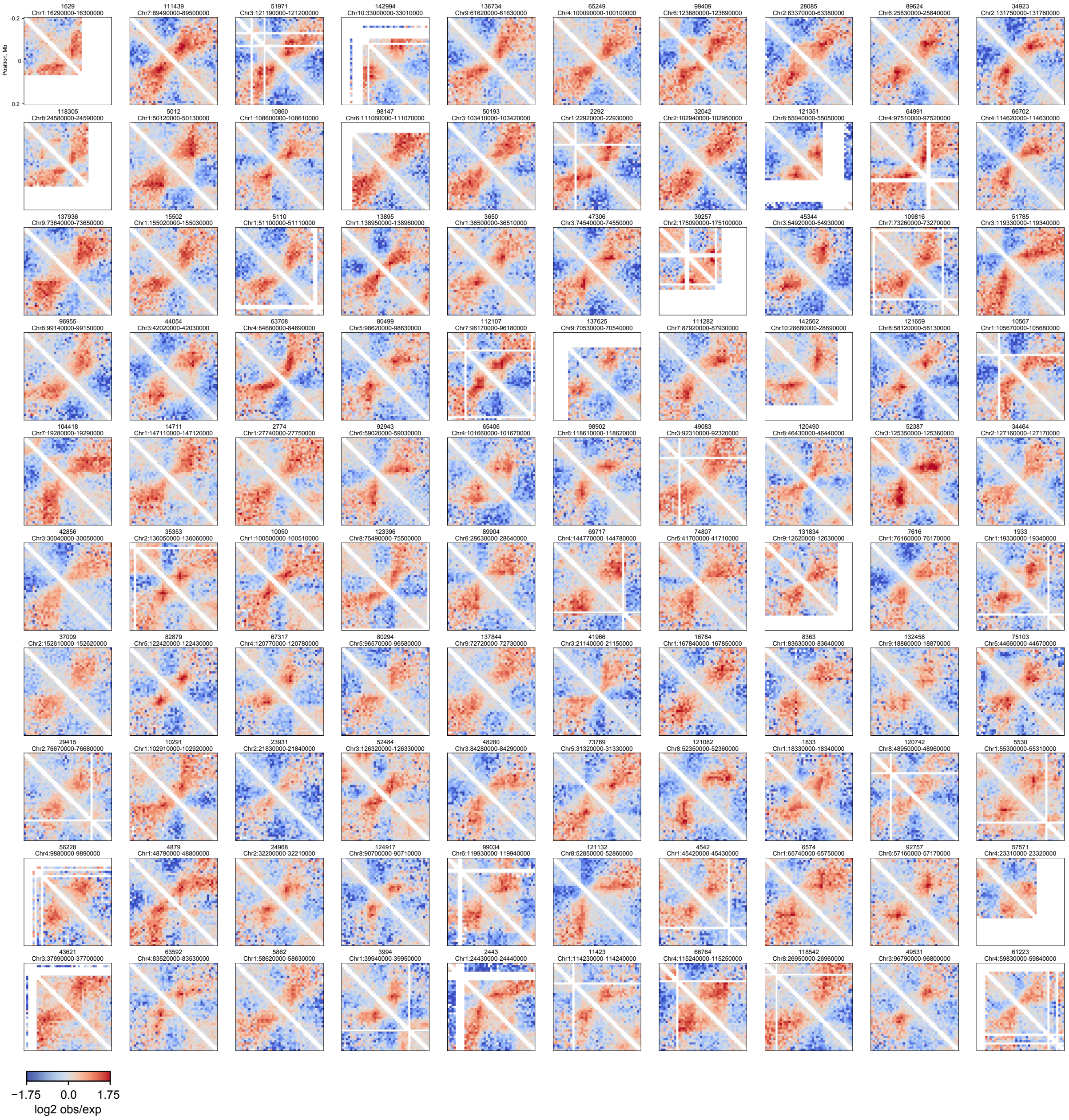

Supplementary Figure 13.

Individual fountains of frog *Xenopus tropicalis* at developmental stage 11.

Snippets of Hi-C maps of 100 fountain bases (top by fountain score with the reference fountain) called with *fontanka*. Each snippet is named by the genomic bin index of the fountain base and the genomic position of the fountain base.

Source data are provided as a Source Data file.

Examples of fountains called at stage 10 *Oryzias latipes*

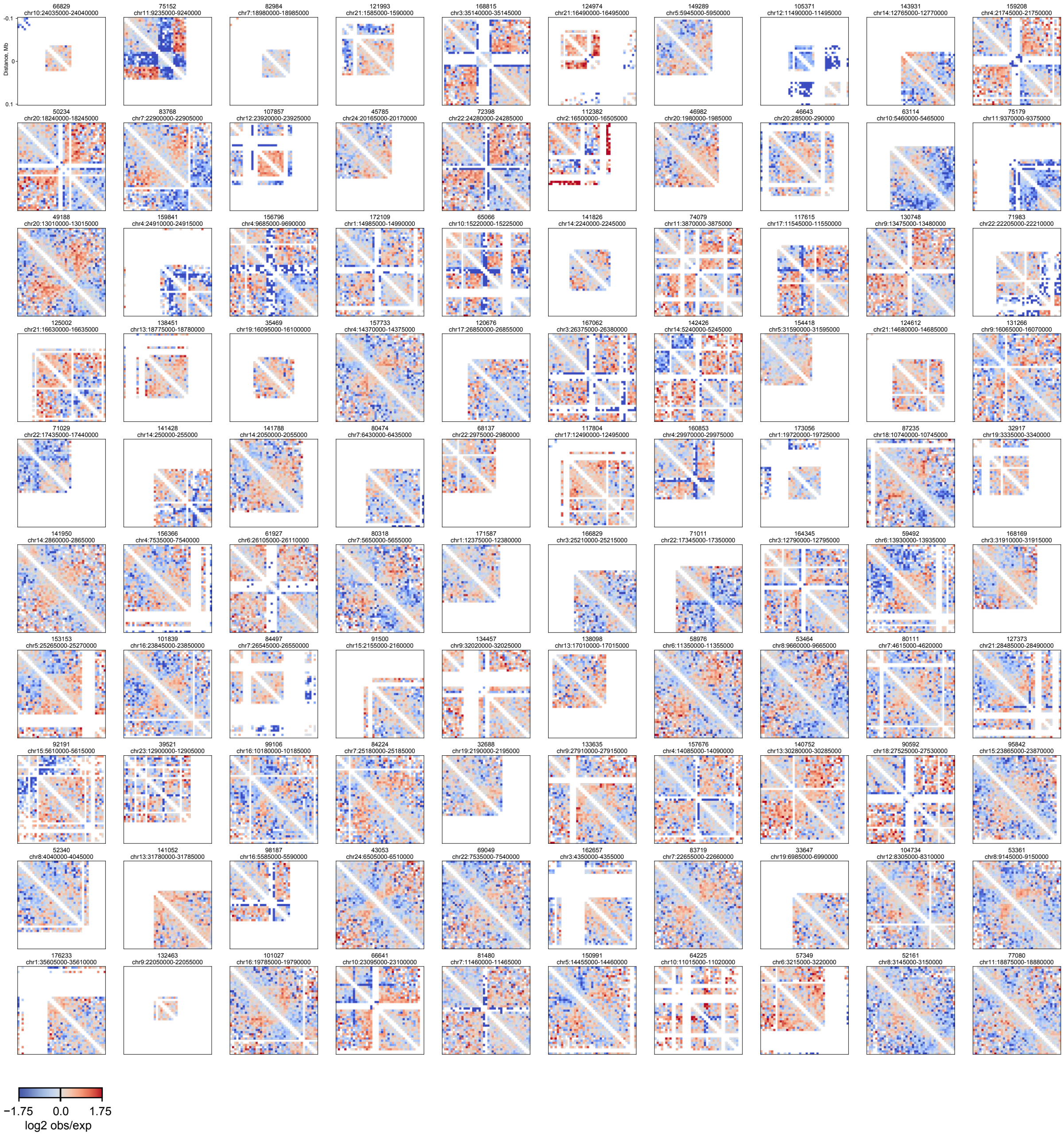

Supplementary Figure 14.

Individual fountains of medaka fish *Oryzias latipes* at developmental stage 10.

Snippets of Hi-C maps of 100 fountain bases (top by fountain score with the reference fountain) called with *fontanka*. Each snippet is named by the genomic bin index of the fountain base and the genomic position of the fountain base. Note that true fountain calls are often contaminated with dots and poorly assembled regions.

Source data are provided as a Source Data file.

Supplementary Methods

Experimental model and subject details

All experiments were performed in accordance with German Animal Protection Law (TierSchG) and European Convention on the Protection of Vertebrate Animals Used for Experimental and Other Scientific Purposes (Strasburg, 1986 [17]). The generation of double and triple mutants was approved by the Ethics Committee for Animal Research of the Koltzov Institute of Developmental Biology RAS, protocol 26 from 14.02.2019. Wild-type fish of AB/TL and mutant strains were raised, maintained, and crossed under standard conditions as described by Westerfield [18]. Embryos were obtained by natural crossing (4 males and 4 females in 1,7 l breeding tanks, Techniplast). Wild-type and mutant embryos from natural crosses were collected in parallel in 10-15 minute intervals and raised in egg water at 28.5°C until the desired stage. The staging was performed following the Kimmel staging series [19]. Stages of the mutant embryos were indirectly determined by observation of wild-type embryos born at the same time and incubated under identical conditions.

Generation of MZ*sn*, MZ*pn* double mutant and MZ*triple* triple mutant embryos and maintenance of the mutant fish lines

Maternal-Zygotic (MZ) homozygous mutant embryos MZ*sn* (MZ*sox19b* ^{m1434}*nanog* ^{m1435}) were obtained in three subsequent crossings. First, MZ*nanog* ^{m1435} [20] homozygous null-mutant males were crossed with MZ*sox19b* ^{m1434} [9] homozygous null-mutant females. The double heterozygous fish were raised to sexual maturity and incrossed. The progeny developed into phenotypically normal and fertile adults. Genomic DNA from tail fin biopsies was isolated and used for genotyping. We first selected *sox19b-/-* homozygous mutants by PCR-with primers Sox19b-f1 5′-ATTTGGGGTGCTTTCTTCAGC-3′ and Sox19b-r1 5′-GTTCTCCTGGGCCATCTTCC-3′. This gives a product of 362 bp length, which contains two restriction sites for BbsI. 5 µl of the PCR mix was digested overnight with 5 units of BbsI (New England Biolabs) in 30 µl volume. The digestion of the wild-type PCR product resulted in three bands with sizes of 40 bp, 132 bp, and 190 bp. In the *sox19b* mutant allele, where one of the BbsI sites was destroyed, the digestion resulted in 40 bp and 322 bp bands. To select the double homozygous fish, we used the genomic DNA from *sox19b-/-* homozygous mutants by PCR with primers Nanog-f1 5′- TCTAAACCCGCCCACAACC-3′ and Nanog-r1 5′-GGTCGGGCTCAGTCTTGTTG-3′, resulting in a 523 bp product. After digestion with *Nde*I (overnight, 37°C), fragments of 284 and 238 bp lengths were generated in the wild-type allele, while the mutant allele was not digested. MZ*sn* embryos for experiments were obtained from incrosses of double homozygous *sox19b*-/-; *nanog*-/- fish. The line was maintained by crossing *sox19b*-/-, *nanog* -/- males with *sox19b*-/-, *nanog* +/- females.

MZ*sn* homozygous mutant embryos were obtained in three subsequent crossings. First, MZ*nanog* ^{m1435} homozygous males [20] were crossed with MZ*spg* ^{m793} [21] homozygous females. *Spg* ^{m793} allele carries an A->G point mutation in the splice acceptor site of the first intron of the Pou5f3 gene, which results in the frameshift starting at the beginning of the second exon, before the DNA-binding domain. Spg ^{m793}

is considered to be a null allele. The double heterozygous fish were raised to sexual maturity and incrossed. To bypass the early requirement for Pou5f3 in the *spg*^{m793} homozygous mutants, one-cell stage embryos were microinjected with 50-100 pg synthetic Pou5f3 mRNA. The fish were raised to sexual maturity (3 months), and genomic DNA from tail fin biopsies was isolated and used for genotyping. We first selected *nanog*-/- homozygous mutants by PCR with Nanog-f2/Nanog-r2 primers, followed by restriction digest with NdeI [20]. To select the double homozygous fish, we used the genomic DNA from *nanog*-/- homozygous mutants to PCR-amplify the region flanking the *spg*^{m793} allele. We used the following PCR primers: spg-f1 5'-` GTCGTCTGACTGAACATTTGC -3' and spg-r1 5'-` GCAGTGATTCTGAGGAAGAGGT -3'. Sanger sequencing of the PCR products was performed using a commercial service (Sigma). The sequencing traces were examined, and the fish carrying A to G mutation were selected. MZ*pn* embryos for experiments were obtained from incrosses of double homozygous *spg*-/-;*nanog*-/- fish. The line was maintained by crossing *spg*-/-, *nanog* -/- males with *spg*-/-, *nanog* +/- females and microinjecting Pou5f3 mRNA in each generation.

To obtain the triple Maternal-Zygotic homozygous mutant embryos MZ*triple*, MZ*sn* double homozygous males were crossed with MZ*ps* (MZ*sox19b*^{m1434}*spg*^{m793} double homozygous females [9]. The *sox19b-/-*; *spg+/-*; *nanog* +/- progeny was raised to sexual maturity and incrossed. The progeny was microinjected with 50-100 pg synthetic Pou5f3 mRNA at one cell stage, raised to sexual maturity, and genotyped as described above. MZ*triple* embryos for experiments were obtained from incrosses of triple homozygous fish. The line was maintained by crossing *sox19b*-/-;*spg*-/-, *nanog* -/- males with *sox19b*-/-;*spg*-/-, *nanog* +/- females and microinjecting Pou5f3 mRNA in each generation.

Genomic DNA isolation and PCR for genotyping

Genomic DNA was isolated from individual tail fin biopsies of 3-month-old fish. Tail fin biopsies or embryos were lysed in 50µl lysis buffer (10 mM Tris pH 8, 50 mM KCl, 0.3% Tween20, 0.3% NP-40, 1mM EDTA) and incubated at 98°C for 10 min. After cooling down ,Proteinase K solution (20 mg/ml, A3830, AppliChem) was added and incubated overnight at 55°C. The Proteinase K was destroyed by heating up to 98°C for 10 min. The tail fin biopsies material was diluted 20x with sterile water. 2µl of was used as a template for PCR. PCR was performed in 25-50 µl volume, using MyTag polymerase (Bioline GmbH, Germany) according to the manufacturer's instructions, with 30-35 amplification cycles.

Zebrafish sperm collection

Adult male fish were anesthetized with Tricain (4% 3-Aminobenzoic acid ethyl ester, pH 6.7) and then positioned with the anal area above. The sperm was taken using a capillary, mixed with 5 µl E400 buffer (9.7 g KCl, 2.92 g NaCl, 0.29 g $CaCl_2$ -$2H_2O$, 0.25 g $MgSO_4$ -$7H_2O$, 1.8 g D-(+)-Glucose, 7.15 g HEPES in 1L dH_2O, pH 7.9) and then with 150µl SS300 buffer (0.37 g KCl, 8.2 g NaCl, 0.15 g $CaCl_2$ -$2H_2O$, 0.25 g $MgSO_4$ -$7H_2O$, 1.8 g D-(+)-Glucose 20ml 1M Tris-Cl, pH 8.0, in 1L dH_2O). The collected sperm was used for nuclei isolation.

Hi-C library preparation

The embryos were obtained from natural crossings in mass-crossing cages (4 males + 4 females). 5-10 cages were set up per genotype, and the eggs from different cages were pooled. The freshly laid eggs

were collected in 10-15-minute intervals. Embryos were incubated at 28.5°C and dechorionated with pronase E (0.3 mg/ml) shortly before the desired stage. 400-600 embryos were homogenized in 2 ml 0.5 % Danieau's with protease inhibitor cocktail (PIC, Roche) and 1 % (v/v) Methanol-free Formaldehyde (Pierce) and fixed for 10 min on a rotating platform. The fixation was stopped with 0.125 M Glycine by shaking for 5 min on a rotating platform. Cells were pelleted on the tabletop centrifuge for 5 min, 500 g, and washed three times with PBST (16mM Na_2HPO_4, 4mM NaH_2PO_4,0.08% NaCl(w/v), 0.002% KCl (w/v), 0.1% Tween 20, pH 7.5), with protease inhibitors. The cells were lysed for 1 min in 1 ml lysis buffer (10 mM Tris-HCl (pH 7.5), 10 mM NaCl, 0.5 % NP-40) on ice. The pellet was washed 2 times with 1 ml ice-cold 1x PBST. In order to count the obtained nuclei, the pellet was resolved in 1 ml ice-cold 1x PBST, of which 10 µl were diluted 1:1 with 12 µM Sytox® green. The nuclei were scored under a fluorescence microscope using the Neubauer counting chamber. The residual nuclei were snap-frozen in liquid nitrogen and stored at -80 °C. 2.5-3 million nuclei were used for one Hi-C experiment according to the published protocol [22].

Chromatin accessibility changes on fountains in MZ*triple*, MZ*spg,* and MZ*nanog* mutants compared to the wild-type

To evaluate the changes in chromatin accessibility on fountains in the absence of Pou5f3, Nanog, or all three zygotic genome activators, we analyzed ATAC-seq data in the respective mutants and the wild-type [8] using the European Galaxy server [23] (Supplementary Fig.ED Fig. 4).

The coverage of ATAC-seq reads at 1460 fountains was scored using *MultiCovBed* (*Bedtools*) in four ATAC-seq replicates of the wild-type, three replicates of MZ*spg* at 4.3 hpf (GEO accession number: GSE188364), two replicates of MZ*nanog,* and four replicates of MZ*triple* at 4.3 hpf (GEO accession number: GSE215956). *Deseq2* [24] was used to normalize the reads and compare chromatin accessibility in each mutant to the wild-type. Each fountain was scored as weakened in the mutant if the log2 fold change to the wild-type was negative with FDR < 5%. As a result of this analysis, 1460 fountains were divided into five groups, as shown in Supplementary Fig.ED Fig. 4a:
I. P - downregulated in MZ*spg*,
II. N - downregulated in MZ*nanog*,
III. T - downregulated in MZ*triple*, not in MZ*spg,* and not in MZ*nanog*,
IV. All - downregulated in MZ*triple*, MZ*spg,* and MZ*nanog*,
V. the rest - not downregulated in three mutants.
Group assignments, Deseq2-normalised ATAC-seq values per replicate, average ATAC-seq values, and the values of log2 fold change in each of the mutants compared to the wild-type are listed in the Supplementary Dataset 2 for all fountains and were used for Supplementary Fig. 4d-g.

GREAT analysis

For predicting the function of cis-regulatory regions within fountains, we used GREAT (Genomic Regions Enrichment of Annotations Tool [25] on all ATAC-seq peaks [8] overlapping fountains. ATAC-seq peaks were associated with genes using a single nearest gene: 50000 bp max extension rule.

Replication timing data analysis

Replication timing data were from [5]. Genomic .txt files containing coordinates in danRer10 (chromosome number in column 1 and chromosomal location in column 2), and replication timing

value in column 3, were obtained from GEO GSE85713. Timing value represents S-phase copy number relative to G1 sample, normalized to a genome mean of 0 and standard deviation of 1. Files were converted to .bedgraph format and danRer11 assembly using the *liftover* tool [26]. Genomic coordinates of the initiation zones were obtained from Chris Sansam. Heat maps and profiles were built using *Deeptools* [27].

Hi-C data mapping

Hi-C reads were mapped with Open2C *bwa-mem* [28] (version 0.7.17-r1188) and *pairtools*[29]-based pipeline *distiller-nextflow* (version 0.3.3). For mapping, we used UCSC's danRer11 assembly with removed unplaced contigs [30]. For parsing, we used the walks policy "all" for parsing pairs [29]. This increased the library pairs yield by parsing all possible pairs from each sequenced read pair. Cool files were created by *cooler* (version 0.8.10), and replicates were merged by summation [31]. Normalization was done by iterative correction [32] with default parameters (minimal count per genomic bin 0, minimum number of non-zero pixels per bin 10, maximum median absolute deviation of pixel values per bin 5, both cis- and trans-contacts included, tolerance 1e-05, and 200 maximum iterations).

Processed coolers from this work are available at GEO [33] https://www.ncbi.nlm.nih.gov/geo/query/acc.cgi?acc=GSE195609. We also re-processed data from previously published zebrafish embryogenesis works [34,35], and made the output available through the Open Science Foundation [36] website: https://osf.io/mt4vf/. Interactive HiGlass views are available at Resgen at https://resgen.io/galitsyna/Zebrafish_embryogenesis/.

Hi-C data processing

Hi-C data visualization

Normalized Hi-C signal was visualized with HiGlass genome browser [11] and resgen [37] (Figure 1a,b, Supplementary Fig 7b, 8a) in log-scales and "fall" colormap.

Centromere positioning in *Danio rerio*

We visualized Hi-C maps for *Danio rerio* in HiGlass genome browser [11] and manually marked the locations of centromeres following the criteria: (i) centromeres must be located far from chromosome ends; (ii) regions around centromeres should have enriched interactions with regions around other centromeres in trans; (iii) centromeres should have unmapped DNA as a sign of centromeric repeats. The beginning and the end of the unmapped DNA served as the centromere start and end, respectively. We later used this annotation to plot the Rabl configuration.

P(s) curves and derivatives

To calculate P(s) curves, also named scalings, for Supplementary Fig. 1d, we used *cooltools* [38] v0.5.4 *expected_cis* at 1 Kb resolution. We removed all Hi-C contacts shorter than 2 Kb, binned contacts in log space to produce evenly sized points in double log coordinates, then smoothed contacts with a Gaussian kernel with sigma 0.05 to even out noisy interactions at the ends of chromosomes and applied aggregation per chromosome arm (defined as in "Centromere positioning"). P(s) derivatives were calculated by taking the gradient at each point of the resulting P(s) plot. For each dataset, the approximate distance ranges of peak locations were manually marked and then validated by searching for local maxima of the derivative plots at distances under 120 Kb.

Note that saddle plots (Supplementary Fig. 1b) and Hi-C data snipping (prior to average pileup and fountain calculation) are separate analyses that also utilize expected Pc(s) (see below). For these analyses, we did not smooth or aggregate P(s) contacts.

Removal of poorly mapped and surrounding genomic regions

While analyzing centromere positions, we noticed that the Hi-C data frequently had rearrangements and poorly mapped regions, noticeable as sharp shapes and edges in the smooth-looking map. Further analysis, thus, required the exclusion of poorly mapped regions. For that, we created a track of unmappable genomic bins (or bad bins).

Firstly, we defined bin as bad if it was impossible to normalize through the default cooler balancing (see criteria in the "Hi-C data mapping") in at least one of the replicate or merged datasets. Secondly, we marked all genomic regions located at least 50 Kb away from these bins as bad because they might be affected by balancing problems due to the absence of short-range interactions. Moreover, bad bins frequently had genomic rearrangements around them, which might affect TAD and fountain calling.

These two criteria resulted in the extended list of 49843 genomic bins subject to removal from most of the Hi-C analyses (out of the total 134526 bins, with 4799 bad bins located solely at chr4).

Building developmental trajectories

To build developmental trajectories, we calculated pairwise SCC (stratum-adjusted correlation coefficient) [39] between all zebrafish Hi-C datasets from previous studies and this work (replicates merged). For that, we used the R library HiCRep [39] with a minimum genomic separation of 0, a maximum separation of 5 Mb, a window size of 3, and a data resolution of 100 Kb. The resulting SCC was input to PCA analysis with *sklearn* [40] (version 1.7.2). The first two PCs jointly explained around 79% of the variance, with a substantial drop at PC3: PC1 47.7%, PC2: 23.1%, PC3: 17.4%, PC4: 4.1% (Fig. 1f). Note that the PCa was done using all zebrafish Hi-C available experiments to us, including Hi-C of mutants. Mutants were excluded from visualization for simplicity of representation.

Average Rabl configuration

To assess Rabl configuration (Supplementary Fig. 1a,c), we plotted average Rabl pileups. We extracted the trans interactions for each pair of chromosome arms at 250 Kb resolution (per-arm snippets). We reshaped per-arm snippets of different sizes into 200x200 squares with *cooltools* [38] (version 0.5.4), which is a common strategy for averaging Hi-C pileups of different sizes [41]. We then averaged all snippets and displayed log2 over expected (average number of trans-interactions).

The centromeric score measures the enrichment of contacts between centromeres of different chromosomes relative to all trans interactions. To calculate the centromeric score, we started with the average Rabl pileup (reshaped and normalized), took a 40x40 window around its center, and calculated the median value.

Compartment calling and saddle plots

For compartment calling (Supplementary Fig. 1b,c), we used *cooltools* (version 0.5.4) cis eigendecomposition with *eigs_cis* [38]. We used cis decomposition and not trans to avoid the influence of the prominent Rabl configuration.

Phasing of the eigenvector is an important step of compartmental calling that defines the sign of the eigenvector with A compartment [32,42]. Phasing of the mammalian first eigenvector is based on correlating the eigenvector with either the number of genes or GC content [32,42,43], because both are

enriched in the A compartment of mammals [38]. For example, in mouse, the first eigenvector at the late 2-cell stage of embryo development [44] highly correlates with both GC content (Pearson corr. 0.64 at 25 Kb bin size) and coverage by genes (Pearson corr. 0.28).

Surprisingly, the first eigenvector of zebrafish Hi-C at 5.3 hpf does not correlate with GC content (Pearson corr. of -0.037 at 25 Kb bin size) and poorly correlates with coverage by genes (P. corr. 0.137). However, the first eigenvector at 5.3 hpf correlates well with replication timing at shield stage [45] (P. corr. 0.59). Thus, we phased the first eigenvector of Hi-C maps with replication timing instead of conventional GC content. 11 hpf first eigenvector correlated the best with bud RT (P. corr 0.65), and 25 hpf first eigenvector correlated the best with RT measured at 28 hpf (P. corr 0.65) [45].

Saddle plots (Supplementary Fig. 1b) were constructed by aggregating observed over expected cis Hi-C signal at 100 Kb (calculated by normalizing balanced Hi-C signal by expected with no smoothing), based on grouping into 10 groups by the first eigenvector for 25 hpf Hi-C experiment. We used *cooltools saddle* (version 0.7.1) module for the computations [38].

Compartment score (Supplementary Fig. 1c) was defined as the mean log2 number of AA and BB interactions in saddle plots minus the mean log2 number of AB and BA interactions. The transition point between A and B compartments was defined by zero of the first eigenvector. For the distribution of compartmental scores, we calculated the compartment scores for each chromosome and plotted the resulting values as distributions.

Insulation score

To capture the general properties of the scalings in embryogenesis, we first calculated insulation scores [46] with *cooltools* [38] (version 0.5.4) at 5 Kb resolutions for each replicate of each developmental stage of zebrafish. We set the window size to 200 Kb. Next, we found local peaks in the insulation score profiles, considered peaks reproducible between replicates as boundaries, and used them for insulation strength characterization in each experiment (Supplementary Fig. 1c).

For insulation profiles (Fig. 2a), we first constructed *bigwig* files with insulation scores with *bioframe* [47] (version 0.8.0) and then aggregated the signal in the genomic windows by *pybbi* [48] (version 0.4.2), as was introduced [49].

TAD calling

For boundary and TAD detection in Fig. 2a and Supplementary Fig. 8c, we performed more rigorous TAD boundary calling. We processed each replicate of each developmental stage with *cooltools*-based and insulation score-based *HiChew* tool [50] at 10 Kb, as explained below. We set the expected TAD size to 200 Kb 34 and automatically assessed the optimal TAD-calling parameters to match the expectation. Next, we removed the TAD boundaries that overlapped with the bad bins. All code for this analysis is deposited in the GitHub repository https://github.com/encent/danio-2022.

Function *hichew.calling.boundaries* produces TAD boundaries, given specific resolution and the expected TAD size parameter. By iterating over the insulation window size grid, it finds, for each chromosome, the insulation window size parameter that minimizes the difference between the user-defined expected TAD size parameter and the median TAD sizes obtained during the grid iteration. The resulting TAD boundaries annotation is calculated using the *cooltools* functions *calculate_insulation_score* and *find_boundaries*, with the insulation window size parameters determined for each chromosome, as explained before. All code for TAD boundaries calling using *HiChew* utility is deposited in the Jupyter notebook https://github.com/encent/danio-2022/blob/main/src/TAD_boundaries_calling.ipynb.

For the confident set of TAD boundaries at 11 hpf in Fig. 2a,b, Supplementary Fig. 2c, we further selected only those boundaries that contain at least one ATAC-seq peak supported by CTCF motif (as in "CTCF binding inference from ATAC-seq" section).

Fountain calling with *fontanka*

Reference fountains

We first randomly selected regions on chromosomes 1 and 2 and manually marked fountains there. In total, we collected 34 fountains from chromosome 1 and 18 fountains from chromosome 2. This fountain set was used as a reference fountain for fountain calling control.

Fontanka protocol

We designed *fontanka*, a *cooltools*[38]-based tool for fountains calling in Hi-C maps (Fig. 1b-c). Although *fontanka* is designed to call any on-diagonal pattern in a Hi-C map, here we applied it to call fountains. Fontanka has four principal steps (Supplementary Fig. 1e):

(i) **Snippets extraction.** *Fontanka* rolls a square window of specified size along the main diagonal of the Hi-C matrix and extracts the observed over expected signal. Each window is assigned to the genomic point of its center.

(ii) **Convolution.** Instead of an insulation diamond window implemented in *cooltools* insulation [38], *fontanka* performs two types of convolution:

(a) Fountain score calculation. Each snippet is convolved with the fountain mask, which produces a fountain score for the corresponding genomic region. In this work, we used the average pileup for all reference fountains as a fountain mask. The convolution score can be based on the summed product of the snippet with a mask, mean square error, and Pearson or Spearman correlation. Here, we used Pearson correlation as a measure for fountain score, which proved to be the best in preliminary tests of the library. Thus, the fountain score can have values between (-1, 1), where 1 is the best correlation with the fountain pattern, and 0 is the absence of correlation.

(b) Noise score calculation. Since Hi-C maps frequently displayed rearrangements relative to the default danRer11 genome, we aimed to filter out potential rearrangements as a noise source in our data. For that, we applied another convolution round with the Scharr operator, approximating the gradient of the Hi-C map [51] [52]. We averaged the Scharr operator by two orientations (vertical and horizontal) for each window pixel, resulting in a Scharr score. The Scharr score measures the noise and sharpness of the signal in the corresponding window. Too noisy or sharp patterns in Hi-C snippets result in high noise scores, while smooth Hi-C maps yield Scharr scores closer to zero.

As a result, each fountain candidate thus has two characteristics: fountain score and noise score (Supplementary Fig. 1f). The higher the fountain score, the more pronounced the fountain-like structure. The smaller the Scharr score, the less noisy the local Hi-C map and the less probable the rearrangements at the corresponding genomic locus.

(iii) **Peak calling.** *Fontanka* detects local maxima of the fountain score by *cooltools find_peak_prominence* function [38]. This function detects peaks in the 1D genomic signal of the fountain score and assigns peak prominence to each one. The resulting peaks were considered candidate fountains (Supplementary Fig. 1e).

(iv) **Thresholding and filtration.** Candidate fountains contain potentially very weak and noisy false calls. In order to minimize the contribution of false calls, we set strict requirements for fountains and

select the most confident set of fountains (Supplementary Fig. 1e).

(a) We first remove the fountains with weak prominence. We noticed that the distribution of the fountain peak prominence is a mixture of two distributions: small noisy calls and long tail of high values of potential true peak calls. To separate those distributions, we applied Li's iterative method for finding the separation point by using the slope of the cross-entropy [53], as implemented in *scikit-learn* [54].

(b) Next, we remove fountains that fall too close (<50 Kb) to the nearest bad bin (see "Removal of poorly mapped and surrounding genomic regions" section of Methods).

(c) We remove all the fountains that have a negative correlation with the fountain mask.

(d) We next require that the fountains detected in the merged dataset are detected in individual replicates with an offset of 20 Kb (on either side of the fountain).

(e) We apply a filter for potential genomic rearrangements and misassembly problems. Genomic rearrangements should be present at all the stages, including 2.75 hpf, which does not have visible non-artifactual on-diagonal patterns in Hi-C. Thus, any fountain detected at 2.75 hpf is probably a result of the erroneous call of genomic rearrangement as a fountain. We removed those fountains at 5.3 hpf that fall within 20 Kb from the nearest constant "fountain" at 2.75 hpf (reproducible between replicates at this time point).

(f) Another filter for potential genomic misassembly problems filters out the top 25% of candidate fountains based on their Scharr score.

Out of 3391 candidate fountains at 5.3 hpf passing filters (iv.a)-(iv.c), 2199 were confirmed in replicates (64.8%, filter (iv.d)). 1110 genomic bins were found as constant "fountains" at 2.75 hpf. Filter (iv.e) resulted in 1947 candidates, out of which 1460 were finally selected for the analysis (iv.f). The final list of fountains with fountain scores and fountain peak scores is provided as Supplementary Dataset 1.

15 out of 34 reference fountains at chr1 (44.1%), and 10 out of 18 reference fountains at chr2 (55.5%) were successfully found by *fontanka* with all filtration steps (Supplementary Fig. 1f).

Since the Open2C software is under ongoing development, we fixed the software versions to *fontanka* v0.2, *bioframe* v0.8.0 [47], *cooler* v0.10.4 [31], and *cooltools* v0.7.1 [38] for fountain calling.

Comparison of fontanka and Chromosight

We noted that the fountain structures are similar to hairpins [55], and that the hairpin-calling algorithm (*Chromosight*) can be applied to call fountains in our dataset. With *Chromosight* v1.6.3 and default parameters, we identified 9 out of 34 reference fountains at chr1 (26.5%) and 7 out of 18 reference fountains at chr2 (38.9%). Notably, *Chromosight* output had an average score of 0.14 and an average pileup that did not resemble the average reference fountain. *Chromosight* results are posted online at https://github.com/agalitsyna/fontanka/blob/master/examples/01_fontanka_vs_chromosight.ipynb.

Comparison of fontanka and the protractor tool

Another algorithm for fountain-like structure calling was proposed for jets [2]. It relies on calculating the contacts at certain distances away from the base (protractor tool). To replicate this approach, we calculated the number of Hi-C interactions normalized by the expected in a line perpendicular to the main diagonal of the Hi-C map for the whole genome. Then, we compared the fountain average protractor versus all non-fountain bins. Indeed, the protractor tool showed enrichment of the contacts at distances up to 150 Kb (Supplementary Fig. 8b). We summed up the protractor tool average over all

distances up to 200 Kb and correlated it with the fountain score (Supplementary Fig. 1g). Both scores correlated with 0.32 Pearson correlation, 0.44 Spearman correlation.

Enrichment of developmental regulatory elements at fountains

For the analysis of regulatory elements at the fountains (Fig. 2c, Supplementary Fig. 2e, Fig. 4g-h), we started with developmental regulatory elements from [6], also called conserved predicted ATAC-seq-supported developmental regulatory elements (cPADREs). Briefly, cPADREs are consensus ATAC-Seq peaks, annotated by ten ChromHMM states at the dome stage. ChromHMM states were obtained from the set of histone modifications measured in *Danio rerio* development in [6].

For the enrichment analysis, we first split the genome into non-overlapping 10 Kb bins. Then, we calculate the coverage of each genomic bin by the regulatory element of a certain type with the *bioframe coverage* tool [47]. Then, for each bin, we calculate the relative enrichment of the regulatory element (the ratio between coverage of the bin by a regulatory element divided by coverage by open chromatin of any type). We then average the enrichments for all bins at the fountain bases.
As a control, we randomize the positions of fountains and calculate the average enrichments again for each state. This procedure preserves the distribution of ATAC-Seq peak sizes and the relative contribution of each state to PADRE annotation. For the FDR calculation, we approximated the control distribution by a Gaussian and calculated the probability of obtaining an observed value or larger.

Hi-C snipping and average pileup

To snip the Hi-C maps (across the whole manuscript), we used *cooltools pileup* function [38]. Pileup takes one or multiple genomic locations and returns a square window of specified size around these locations.

To build average pileups over genomic loci (e.g., fountains), we took the mean of corresponding pixels of all windows snipped around fountains.

Differential fountains in MZ*triple*

To define the fountains that go down, stay unchanged, or go up in MZ*triple* (Supplementary Fig. 3e), we first calculated the fountain scores by fontanka (as described above). Then, we calculated the difference in fountain scores between WT and MZ*triple* for each genomic bin. We then plot the distribution of all differences and fit it using the Gaussian distribution (by *scipy stats norm* [14] function). We then defined DOWN fountains as those that have a drop in fountain score more than 1.5 standard deviations from zero and UP fountains as those that have a growth of fountain score more than 1.5 standard deviations from zero. The rest of the fountains were considered unchanged.

ChIP-Seq data analysis

We re-analyzed ChIP-Seq data from [3,10,35,56,57] with *chipseq-nf* 1.2.1 with default parameters [58]. The processing mode was set either to single-end or paired-end based on raw sequencing annotation in the SRA database (see Supplementary Dataset 6).

CTCF binding inference from ATAC-seq

Unfortunately, at the time of this research, CTCF ChIP-Seq data are not available for most stages of development of zebrafish that we study (except one dataset for 10 hpf from [3]). However, multiple

ATAC-Seq profiles are available from the Danio Code database [59]. This allowed us to infer CTCF binding from ATAC-Seq data by assuming that the ATAC-Seq peak with the strong CTCF motif has CTCF bound at this stage.

First, we called CTCF motifs in the danRer11 genome. Generic vertebrates CTCF motif (JASPAR MA0139.1) instances were called by JASPAR whole-genome *PWMScan*-based [60] motif scanner [61]. The background GC content was set to 0.317 (A and T frequencies) and 0.183 (G and C). We used a p-value threshold of 5e-02 and an r threshold of 0.8.

Next, we downloaded ATAC-Seq peaks from the DCC database [59] in narrow peak format for 4.5 hpf (DCD019127DT) and 12 hpf (DCD019077DT). Each ATAC-Seq peak was marked as CTCF-bound if it had at least one CTCF motif.

The selection of parameters for motif calling was made by benchmarking against CTCF ChIP-Seq peaks obtained at 10 hpf from [3] (re-processed with *chipseq-nf* with default parameters [62]).

For coverage pileups in Supplementary Fig. 2c, we stored the CTCF-containing peaks with their abundance as bigwig files with *bioframe* [47] and then aggregated the signal in the genomic windows by *pybbi* [48].

Simulations of loop extrusion

Simulations of loop extrusion (Fig. 5, Supplementary Fig. 5-6) were based on the *OpenMM*-based [63] (version 8.0) Python library *Polychrom* [64] (version 0.1.1). We split it into five steps: (1) 1D simulation of extruders movement on the genome, (2) 3D simulation of polymer, (3) *in silico* reconstruction of interaction probabilities, (4) assessment of goodness of fit of the simulations to real data, (5) parameter sweep of the model.

The implementation is available at https://github.com/agalitsyna/polychrom_workbench under the *targeted_extrusion* directory.

1D simulation

We simulated the translocation of extruders on a 1D lattice [65], where each point represented a genomic position. Each position can either (1) have a regular role or (2) serve as a loading platform for the targeted extruder. For regular positions, we assumed uniform probabilities of loading and unloading. For loading platforms, we set up the probability of cohesin landing increased by the enrichment factor.

To simplify the model, we assumed (1) immediate reloading of unloaded cohesin, and (2) separate pools of extruders that can load at regular positions versus loading platforms. Before running the simulations, we ensured that cohesins were fully loaded on a lattice.

As in our previous work [65], simulations were organized in rounds, with all cohesins performing translation with both legs in opposite directions at each round. Each simulation lasted for 10 010 000 translocation rounds, and we sampled each 10th step as a readout of 1D simulation. We removed the first 10000 steps to ensure that we sample the steady state of loop formation and not the earlier stages of loop formation and maturation [66].

The 1D lattice size was 50000 monomers, with each monomer corresponding to 1 Kb of DNA (thus, the whole simulated molecule was 50 Mb).

The size of the loading platform was set to 1 monomer (1 Kb), and the platforms were distributed each 500 Kb to correspond to the mean distance between fountains in Hi-C data. Technically, we

generated 20 groups of the size 2500 to allow averaging over multiple groups in a single conformation when calculating the average fountain, as we proposed previously [67].

For the basic simulations of targeted loading, we assumed that there are (1) no boundaries that stall cohesin legs, (2) only other cohesins can stall the legs of cohesins, (3) legs of cohesin (if not stalled) are moving simultaneously, (4) a free leg of cohesin with another stalled leg can move.

For simulations with random barriers, we assumed that (1) cohesins load only at the loading platforms, (2) random barriers are placed at each genomic position in a 100 Kb window around the platform, (3) random barriers stall the cohesin leg with some probability (best fit to real data obtained for the stalling probability of 0.005, see Fig. 5c, *middle*).

For simulations with decoupled legs of cohesin, we assumed that (1) cohesins load only at the loading platforms, (2) the probability of stepping is set for each leg independently (best fit to real data obtained for the probability of stepping set to 0.99, see Fig. 5c, *right*). We note, however, that by decreasing the probability of stepping, we also decreased the speed of extruders, which may explain why we obtained the stepping probability close to 1.

3D simulation

As in our previous works [66–68], we represented chromatin as a polymer with spherical monomers connected by harmonic bonds with stiffness and soft-core repulsive potential. Simulations were performed with a variable Langevin integrator in the periodic boundary conditions [67] with a volume density of 0.1. For simulations and setting up the bonds, we used the OpenMM-based [63] Python library Polychrom [64]. A harmonic bond connected the two monomers held by the extruder. The number of 3D-simulation time steps per 1D simulation step was set to 200 [69].

In silico reconstruction of interaction probabilities

To calculate contact maps, we recorded all the pairs of monomers located closer than 5 monomer radii. We then averaged the interactions over multiple equivalent groups to obtain a simulated 2.5 Mb region with fountains. To obtain a simulated average fountain at 10 Kb resolution, we snipped 200 Kb windows around the loading platforms and coarse-grained them by a factor of 10. We then normalized the snippets by the expected.

Goodness of fit of the simulations to real data

The goodness of fit was assessed by the correlation of the simulated average fountain with the Hi-C average fountain. For that, we compared each *in silico* Hi-C of the simulated fountain with an average reference fountain. As a quality measure, we calculated (1) the Spearman correlation between matrices of average fountains, (2) protractor sections of average fountains, and (3) cross-sections of fountains at characteristic distances from diagonal (Sketch: Supplementary Fig. 5c, results: Fig. 5d-e, Supplementary Fig. 5b-c, more illustrative examples for different simulation parameters: Supplementary Fig.. 6). Both matrices were taken at 10 Kb resolution, and *in silico* Hi-C matrices were coarse-grained for this purpose (as opposed to non-coarse-grained visualizations of average simulated pileups, which are displayed at 1 Kb resolution).

For additional validation, we checked the interactions between loading platforms (fountain bases) by snipping off-diagonal in silico Hi-C matrices and verified that there was no enrichment of interactions (Supplementary Fig. 5d).

Parameter sweep

The simulations were organized as a set of Python scripts: 1D extrusion, 3D simulations, and 2D contact map construction. For the 1D extrusion, we could vary lifetime, separation, and the level of targeted loading. For 3D simulations and 2D contact map construction, we fixed the parameters. To automate the parameter sweep, we implemented a snakemake [70] pipeline combining all three steps. Tested parameters and the resulting goodness of fit are presented in Fig. 5d (mean square error (MSE) of the protractor) and Supplementary Fig. 5b (Spearman correlation coefficient).

Supplementary Notes

I. Fountain-like structures in other biological systems

We define fountains as *patterns of contacts that emanate from a single genomic locus and broaden with distance from the diagonal.* The fountain is a novel pattern of chromatin organization, which is as widespread in zebrafish embryogenesis as TADs, stripes, and dots are in, for example, differentiated mammalian cells [67,71]. We acknowledge that fountains and fountain-like structures might be present in other biological systems, and the mechanisms of their formation might be different from the targeted cohesin extrusion that we propose here.

On the whole-chromosome scale, *Rabl configuration* of some eukaryotes [72,73] and *hairpins* in bacteria [74–76] resemble fountains. Both patterns represent the arms of chromosomes aligned with each other.

Rabl configuration is a consequence of the memory of the mitotic organization, where chromosomes in interphase keep the alignment similar to that in the mitotic spindle. That mechanism, supposedly, does not require tethering of the arms or targeted loading of the extruder [72]. Although Rabl configuration is prominent in zebrafish (as we and others [35] report), it is unrelated to the relatively small (<200 Kb in length) fountains at 5.3 hpf.

The alignment of chromosomal arms in bacteria, or *hairpin*, results from the loading of SMC complexes by ParB protein at a centromeric locus with parS sites. This system was thoroughly explored through simulations of targeted loop extrusion [75]. However, these findings cannot be transferred to zebrafish, where multiple, much smaller, fountains are scattered throughout multiple chromosomes (Fig. 1b,c, Supplementary Fig. 9a).

Other previously reported fountain-like structures at smaller scales are *local interaction patterns (LIPs)* [77], *flares* [35], *plumes* [78], and *jets* [2] (see Supplementary Table 1 below).

Induction of double-stranded breaks (DSBs) in *S. cerevisiae* leads to the formation of *local interaction pattern (LIP)*, enriched interactions emanating from the DSB. LIPs are 25-Kb in size, and depend on homologous recombination factors, but not cohesin (although cohesin accumulates at their bases). The mechanism of LIPs formation might be, thus, unrelated to active extrusion.

The chromatin of zebrafish sperm cells is folded into at least 333 *flares* [35]. These structures are specific to sperm cells and are absent at the same positions in developing embryos [35]. Visual inspection of the reported Hi-C maps suggests that flares in sperm cells span from hundreds of thousands to millions of nucleotides. These scales are still much larger than we observe for the fountains here. Interestingly, although H3K27ac was enriched at flare bases, Smc3 was not [35]. Thus, the formation mechanism might differ from the targeted extrusion proposed here.

Upon degradation of both Wapl and CTCF in mouse embryonic stem cells (mESCs), the regions with high openness form *plumes* [78]. Like fountains in zebrafish, plumes are transient (appear at 6 hours after induction of degradation and disappear at 96 hours). The bases of plumes are enriched in Rad21 in untreated mESCs and lose this enrichment in Wapl/CTCF degradation [78]. Moreover, plumes disappear upon Rad21 degradation [78], suggesting they are formed by active extrusion by cohesin.

Primary mouse DP thymocytes have at least 38 *jets* of 1-2 Mb in size in wild-type cells [2]. Jets depend on Rad21 and become longer upon CTCF knockout [2]. Simulations of targeted loop extrusion from Guo et al. [2] qualitatively explain the jets' formation. For example, jets get more dispersed with the distance from the diagonal, suggesting that desynchronization of cohesin arms is required for their proper shaping, as we find for fountains in this work. The simulations in Guo et al. [2] assume the desynchronization of cohesin arms as an intrinsic property of cohesin. However, they do not explore

alternative scenarios for the shaping of the dispersed form of fountains/jets.

**System and condition of fountain-like structures appearance**	**Fountain basis**	**# of fountains per genome**	**# of fountains per 1 Mb of genome (if relevant)**	**Reference**
DNA double-strand break formation in *S. cerevisiae*	Double-strand breakpoint	1	-	Piazza et al., 2021 [77]
Zebrafish sperm cells	H3K27ac-enriched regions	333	~0.26	Wike et al., 2021 [35]
Mouse cells with Wapl + CTCF degron	Open chromatin regions (OCIs, defined as high-ATAC-seq-region flanked by low-ATAC-Seq)	852	~0.3	Liu et al., 2025 [78]
Small DP thymocytes	Open chromatin regions, also enriched in H3K27ac	38	~0.01	Guo et al., 2022 [2]
Nematode *C. elegans*	Active enhancers, enriched in cohesin	1263	~13	Lüthi et al., 2023, preprint [79]
Nematode *C. elegans*	Active enhancers, enriched in cohesin	287	~2.9	Kim et al., 2025 [80]
Zebrafish embryos upon ZGA	Open chromatin regions enriched in H3K27ac, H3K4me1, and pioneering factors binding	1460	~1.13	This work
Fusarium graminearum	biosynthetic gene clusters	511 in putrescine and 297 in NaNO3 media	~14 and 8, correspondingly	Shao et al., 2024 [81]
Arabidopsis thaliana	cohesin-enriched regions	1361	~11	D. Wang et al., 2024 [82]

Supplementary Table 1.
Fountain-like structures reported in the literature. Genome size was the sum of all chromosome lengths (as reported by UCSC Genome Browser [83]), excluding mitochondrial chromosomes.

Critically, Guo et al. [2] do not define their extrusion dynamics and length scales in physical terms. For example, the loading platform for cohesin is set to either 100 (“narrow” platform) or 1000 (“broad” platform) monomers (arbitrary units and not genomic coordinates). From the visual comparison of simulated and Hi-C maps in this work, one could assume that one monomer corresponds to 800 bps (the average window analysis for jets is 4 Mb for Hi-C versus 5000 monomers for simulations). Thus, the “narrow” platform in these simulations is, in fact, 80 Kb, which is much larger than what we report for fountains in zebrafish (10 Kb platform, single monomer). Finally, in contrast to Guo et al. [2], our fitting simulation parameters to the Hi-C data allow us to estimate a cohesin processivity of 150 Kb at 1 Kb/s speed and enrichment of targeted loading by a factor of 10.

Finally, consistent with the cohesin model of fountains formation, Lüthi et al. [79] observe 1263 fountains and Kim et al.[80] observe 287 fountains at active enhancers of nematode *C. elegans*, and demonstrate that fountains are cohesin-enriched and depend on cohesin.

Some works report fountain-like structures at the average pileups, which might also represent fountain formation in chromatin. For example, Murine Endogenous Retroviral Elements with a leucine tRNA primer binding site (MERVL) produce an average Hi-C fountain in embryogenesis (late 2-cell and 8-cell, bioRxiv preprint [84]). Interestingly, Micro-C also reveals a fountain-like average at the binding sites of Nanog and, to a lesser extent, Med12 in mESC [85]. However, in both these works, the fountain-like pattern emerges as an average and might be a consequence of averaging centers of TADs, as we show in Supplementary Note III "Limitations of fountain detection as an average pileup" below.

Interestingly, Micro-C can reveal the features of the average fountain at the resolutions of up to 200 bps, which is much below the resolution possible in Hi-C analysis (10 Kb in this work) [85]. For example, fountain in mouse cells have enriched interactions from ~10 Kb to ~100 Kb (just 10 pixels at 10 Kb and 20 pixels at 5 Kb resolution), which can be analysed in greater detail with nucleosomal resolution of Micro-C. Since the fountains might have intricate inner structure, we anticipate that further studies of individual fountains with Micro-C will shed new light on the mechanism of their formation.

Finally, fountain-like structures have also been reported as an outcome of the biophysical modeling of chromatin.

Goychuk et al. [86] model sequence-dependent correlated active forces in chromatin and observe fountain-like structures concomitant with compartment formation. Fountain-like structures originate from small active regions by spontaneous loops by a local hot spot of activity.

Brahmachari et al. [87] observe fountain-like structures in the models with temporally correlated active forces. Fountains are formed at the elements with correlated motion, and become stronger with increased correlation.

Although both these models do not explicitly include loop extrusion (a non-equilibrium process [88]), they might represent the same class of models with non-equilibrium mechanisms. Further work is needed to establish whether fountain-like structures in chromatin are a common signature of non-equilibrium processes in chromatin.

II. Detailed characterization of zebrafish fountains

Overview and visualization. To better understand the genomic context and internal structure of fountains, we inspected representative fountain loci by plotting Hi-C contact maps together with genomic and epigenetic tracks in the same coordinate system (Supplementary Fig. 9a). This joint visualization highlights two recurring features. First, fountains are centered on a well-defined "base" region where contact enrichment originates in the Hi-C map. Second, the fountain base is typically accompanied by hallmarks of regulatory DNA: accessible chromatin, enhancer-associated histone marks, and enhancer-like ChromHMM annotations in the surrounding region. Consistent with our model that fountains are generated by cohesin-driven loop extrusion initiated at these sites, cohesin (Rad21) ChIP-seq signal is enriched at or near the fountain base.

Shape of fountains. We quantified fountain shape by plotting protractor representation of the average fountain and compared it to the non-fountain regions of zebrafish genome (Supplementary Fig. 9b).

Fountains and TADs. Because topologically associating domains (TADs) are typical features of vertebrate Hi-C maps, we also tested whether fountains could be trivially explained as the centers of TADs. To do so, we compared fountain bases distributions around TAD centers (Supplementary Fig. 9c). Fountains are not enriches specifically at TAD centers and are deleted at TAD boundaries, demonstrating that fountains are not simply centers of the TADs.

Epigenetic and regulatory landscape at fountains. We next asked which chromatin features are most enriched at fountain bases during the onset of ZGA around 4-5 hpf (Supplementary Fig. 9d). We compiled epigenetic and transcription-factor datasets available at these stages and compared their distributions at fountain bases to control non-fountain 10 Kb bins. We ranked the factors by the significance of the difference in medians between fountains and control. This analysis shows that fountains are strongly associated with enhancer-like activity. Signals enriched at fountain bases include H3K27ac, PolII binding, chromatin openness, Nanog and Sox2 binding, Rad21 enrichment, H3K4me1, Pou5f3, and p300. In contrast, H3K4me3 (typically associated with promotors) is not significantly different at fountains than in other genomic locations. Notably, CTCF is significantly depleted at fountain bases, reinforcing that fountains are not defined by canonical CTCF-anchored loop features and motivating the hypothesis that their formation does not require CTCF binding at the base.

What changes in mutants explain changes in fountain strength? To connect fountain structure to regulatory state, we asked which chromatin changes best predict fountain weakening in the pioneer-factor triple mutant MZ*triple* (Supplementary Fig. 9e). For each fountain, we calculated the change (delta) of fountain score between wild-type cells and MZ*triple*. We next correlated fountain score changes with the changes for epigenetic factors between wild-type and MZ*triple* [10]. The strongest correlations were observed for changes in ATAC-Seq, H3K27ac, and H3K4me1, indicating that loss of chromatin accessibility and enhancer activation is tightly coupled to loss of fountain strength. By contrast, change in H3K4me3 shows near-zero correlation, arguing against a promoter-centered origin of fountains.

Relationship between fountains and zygotic transcription. Finally, we tested whether fountains are positioned near zygotically activated genes and whether this association depends on transcriptional activity (Supplementary Fig. 10). Using 10 Kb windows centered on 1,460 fountains,

we tested whether fountain-proximal bins overlap transcription start sites (TSSs) of zygotic genes more often than matched control bins sampled within ±1 Mb of each fountain (excluding fountain overlaps). Assigning genes to bins by overlap within ±55 Kb of the TSS, we observe a significant enrichment of zygotic TSSs at fountains compared to controls, indicating that fountains are non-randomly positioned near zygotic genes (Supplementary Fig. 10a).

We then asked whether transcriptional output modulates this relationship. Stratifying zygotic genes by expression level (EBI Expression Atlas; excluding putative maternal transcripts), we found that fountains accumulate preferentially within ~100 Kb of highly expressed genes, exceeding the expectation from randomly sampled genomic regions (Supplementary Fig. 10b). Conversely, when the analysis is reversed (gene categories as a function of distance to fountains), highly expressed genes are enriched near fountains, whereas weakly expressed or silent genes are depleted within ~50-100 Kb distance (Supplementary Fig. 10c). Together, these analyses show a bidirectional relationship: fountains tend to form near active zygotic promoters, and active zygotic genes preferentially reside in the vicinity of fountains, consistent with fountain formation being linked to early transcriptional activation.

III. Limitations of fountain detection as an average pileup

An *average pileup* is a commonly used instrument for Hi-C/Micro-C and other capture techniques analysis [1,38,41,89]. Average pileup relies on averaging fragments of Hi-C maps (*snippets*) between different genomic locations. This tool has been widely applied in comparative studies between different treatments and conditions [90] or between different genomic locations within a single Hi-C/Micro-C map [85]. However, the resulting average is not representative of the conformation of a single genomic locus. It is important to avoid potential misinterpretation of average pileups.

Fountains are the predominant features of Hi-C maps after ZGA (5.3 hpf) of zebrafish embryos, enabling us to perform genome-wide fountain calling with *fontanka*. With *fontanka*, we call individual genomic locations bearing the fountain signature in the surrounding ±200 Kb of the Hi-C map. Visual inspection confirms that snippets have fountains, except for several false positives (Supplementary Fig. 11a). As expected, when averaged, individual fountains produce the average fountain structure with a large fountain score (Supplementary Fig. 11b-c).

However, the average fountain does not guarantee the presence of fountains at individual loci of chromosomes. Individual genomic loci that look nothing like fountains may produce the appearance of an average fountain-looking structure at the average pileup. For example, centers of TADs at 11 hpf average to the fountain-looking structure on a pileup (Supplementary Fig. 12a). This property arises because (1) the centers of TADs have enrichment of contacts between the surrounding regions, and (2) TAD boundaries are located at random distances, washing off the average enrichment nearby. While individual snippets of the pileup do not resemble fountains (Supplementary Fig. 12a), an average pileup looks like a fountain (Supplementary Fig. 12b). This effect is visible only when a large number of genomic positions with TADs of different sizes is averaged.

To avoid this issue, we recommend two strategies: (1) inspecting individual snippets visually for the presence of the fountains (as in Supplementary Fig. 12a) and (2) sampling the individual snippets to the batches and average over limited number of candidate fountains (Supplementary Fig. 12c). Average pileup for the sampled set of candidate fountains can reveal presence of bright dots in the average pattern suggesting that some fountains might in fact be corner peaks of TADs. In summary, we suggest to confirm the presence of the fountains in Hi-C maps at the level of individual genomic loci.

IV. Realistic simulations of enhancer-targeted cohesin loading with CTCF barriers

To analyze the enhancer fountain signature observed in mouse embryonic stem cells (mESCs) and its reorganization upon CTCF depletion, we compared experimental Micro-C pileups [15] to polymer simulations designed to mirror the local genomic context of enhancers and CTCFs in the mouse genome. The main goal of this note is to explain why fountains become more extended but weaker when CTCF is removed, and to test whether this behavior follows naturally from an extrusion model in which cohesin is preferentially loaded at enhancers and is halted by CTCF.

First, we computed average Micro-C pileups centered on enhancers in untreated mESCs and in a matched dataset after CTCF degradation [15] (Fig. 6b and Supplementary Fig. 8a). In untreated cells, the enhancer-centered signal is relatively compact, with stronger interaction intensity concentrated at shorter genomic distances. After CTCF degradation, the average signal becomes more extended, while the overall intensity decreases. We quantified these differences using two summary readouts applied throughout the manuscript (Supplementary Fig. 8a): (i) a protractor that reports the angular distribution and extent of off-diagonal signal in the pileup, and (ii) cross-section at a fixed genomic distance (here, 200 Kb), which capture changes in the peak intensity and spread. Together, these measurements indicate that CTCF loss does not eliminate the enhancer-centered pattern but reshapes it into a longer-range, lower-intensity signature.

Next, we simulated loop extrusion *in silico* using the same set of extrusion parameters as the best-fitting zebrafish simulations. To create a realistic setup, we explicitly incorporated two genomic inputs:
1. Facilitated loading sites at enhancers. We used mESC enhancers [16] as facilitated loading sites.
2. CTCF barriers with orientation. We placed oriented CTCF sites using CTCF peaks from the mESC Micro-C study [15], with orientations inferred by motif calling as in Supplementary Methods "CTCF binding inference from ATAC-seq". These oriented sites were implemented as directional barriers that can stall extruders.

To ensure that the simulated contexts match the distribution of CTCF around enhancers, we selected n=100 random enhancers and, for each enhancer, collected the surrounding CTCF sites (positions and orientations) to define their locus-specific barrier landscape. We then simulated ensembles of such loci and generated average Hi-C pileups centered on the enhancer loading sites, directly comparable to experimental enhancer-centered pileups.

To mimic the presence versus absence of CTCF barriers, we varied the probability of extruder stalling upon encountering a CTCF barrier: (i) strong CTCF stalling probability 0.25**,** (ii) weak CTCF stalling probability 0.01. This approach captures the functional consequence of CTCF depletion in the extrusion model: extruders that would normally be halted near the loading site by CTCFs are instead able to continue extruding over longer genomic distances.

Average simulated pileups at enhancer loading sites reproduce the qualitative trends observed in mESC With strong CTCF stalling (0.25), extruders frequently stall at the first CTCF barriers encountered near the enhancer. This restricts extrusion range and concentrates contacts near the diagonal, yielding a stronger, shorter-range enhancer-centered fountain. In these simulations, the surrounding CTCF sites also generate CTCF-dependent dots (Supplementary Fig. 8b, bottom), often producing a grid-like pattern of enriched interactions between barrier-defined positions. Notably, these

dots can visually dominate the pileup and partially overlay the fountain pattern of single-locus *in silico* Hi-C (Supplementary Fig. 8b, bottom). With weak CTCF stalling (0.01), extruders are minimally impeded by CTCF, so they travel farther from the enhancer loading site before stopping. This disperses contacts over a larger genomic range, producing a fountain signature that is longer but lower in intensity. The protractor reflects this change as a more extended distribution with weaker interaction density, and the 200 Kb cross-section shows a reduction in the central peak height (Supplementary Fig. 8b, right).

These effects are also evident at the single-locus level. For the same genomic locus (example on chromosome 8), the fountain appears more clearly when CTCF-dependent dots are reduced (weak stalling, Supplementary Fig. 8b, second left), whereas strong stalling produces prominent dot patterns that can obscure the underlying fountain. This provides an intuitive explanation for why the fountain can become easier to recognize when CTCF barriers are absent, even though the overall signal becomes weaker.

These effects may contribute to the fact that we do not readily observe fountains at the individual loci of zebrafish chromatin organization at the later developmental stages, where CTCF concentration increases with development and CTCF insulation and dots become more visible (Fig. 1e).

V. Fountain calling in medaka and *Xenopus*

The fountain calling in medaka and *Xenopus* was performed with the same algorithm, fontanka, as in zebrafish with several modifications. The resulting average pileups are presented in Figure 1g,h, and individual examples in Supplementary Fig. 13 and 14.

First, we re-mapped the data from Niu et al., 2021 [91] (*Xenopus tropicalis*, genome xenTro10) and Nakamura et al., 2021 [35] (*Oryzias latipes*, medaka fish, genome oryLat2) with *bwa-mem* [28] and Open2C *pairtools*[29]-based pipeline *distiller-nextflow* version 0.3.3 and walks policy “all”. With the visual inspection of Hi-C maps, we confirmed the presence of fountain patterns at 5 Kb resolution for medaka at developmental stage 10 and 10 Kb resolution for *Xenopus* at developmental stage 11. We filtered out poorly mapped genomic regions at these resolutions (as was done for zebrafish) and ran *fontanka* with the reference fountain mask from zebrafish. We then filtered out fountain peaks that were too weak and had large noise scores (filters iv.a, iv.b, iv.f from “Fontanka protocol” section of Supplementary Methods).

Compared to zebrafish (Supplementary Fig. 11), dot patterns and genomic misassemblies more frequently contaminated the resulting fountains in *Xenopus* and medaka (Supplementary Fig. 13-14). This can be attributed to (1) worse quality of the genome assemblies for these species than for zebrafish; and (2) the presence of dots and TADs at selected developmental stages alongside the fountains.

Despite these discrepancies, the average pileups computed from the final call sets show a clear fountain signature (Fig. 1g,h), and inspection of individual loci confirms that many snippets exhibit fountain-like structure (see Supplementary Note III. “Emergence of a fountain as an average pileup”).

References

1. Gassler, J. *et al.* A mechanism of cohesin‐dependent loop extrusion organizes zygotic genome architecture. *EMBO J.* **36**, 3600–3618 (2017).
2. Guo, Y. *et al.* Chromatin jets define the properties of cohesin-driven in vivo loop extrusion. *Mol. Cell* **82**, 3769–3780.e5 (2022).
3. Meier, M. *et al.* Cohesin facilitates zygotic genome activation in zebrafish. *Development* **145**, (2018).
4. Danio-code dcc. https://danio-code.zfin.org/daniocode/.
5. Siefert, J. C., Georgescu, C., Wren, J. D., Koren, A. & Sansam, C. L. DNA replication timing during development anticipates transcriptional programs and parallels enhancer activation. *Genome Res.* **27**, 1406–1416 (2017).
6. Baranasic, D. *et al.* Multiomic atlas with functional stratification and developmental dynamics of zebrafish cis-regulatory elements. *Nat. Genet.* **54**, 1037–1050 (2022).
7. White, R. J. *et al.* A high-resolution mRNA expression time course of embryonic development in zebrafish. *Elife* **6**, (2017).
8. Riesle, A. J. *et al.* Activator-blocker model of transcriptional regulation by pioneer-like factors. *Nature Communications* (2023) doi:10.1038/s41467-023-41507-z.
9. Gao, M. *et al.* Pluripotency factors determine gene expression repertoire at zygotic genome activation. *Nat. Commun.* **13**, 788 (2022).
10. Miao, L. *et al.* The landscape of pioneer factor activity reveals the mechanisms of chromatin reprogramming and genome activation. *Mol. Cell* **82**, 986–1002.e9 (2022).
11. Kerpedjiev, P. *et al.* HiGlass: web-based visual exploration and analysis of genome interaction maps. *Genome Biol.* **19**, 125 (2018).
12. Zhang, H. *et al.* Chromatin structure dynamics during the mitosis-to-G1 phase transition. *Nature* **576**, 158–162 (2019).
13. Dogan, N. *et al.* Occupancy by key transcription factors is a more accurate predictor of enhancer activity than histone modifications or chromatin accessibility. *Epigenetics Chromatin* **8**, 16

(2015).

14. Virtanen, P. *et al.* SciPy 1.0: fundamental algorithms for scientific computing in Python. *Nat. Methods* **17**, 261–272 (2020).
15. Hsieh, T.-H. S. *et al.* Enhancer–promoter interactions and transcription are largely maintained upon acute loss of CTCF, cohesin, WAPL or YY1. *Nat. Genet.* **54**, 1919–1932 (2022).
16. ENCODE Project Consortium *et al.* Expanded encyclopaedias of DNA elements in the human and mouse genomes. *Nature* **583**, 699–710 (2020).
17. Iii., 18. European convention for the protection of vertebrate animals used for experimental and other scientific purposes. https://rm.coe.int/168007a67b.
18. Westerfield, M. A guide for the laboratory use of zebrafish (Danio rerio). *(No Title)* (2000).
19. Kimmel, C. B., Ballard, W. W., Kimmel, S. R., Ullmann, B. & Schilling, T. F. Stages of embryonic development of the zebrafish. *Dev. Dyn.* **203**, 253–310 (1995).
20. Veil, M. *et al.* Maternal Nanog is required for zebrafish embryo architecture and for cell viability during gastrulation. *Development* **145**, (2018).
21. Lunde, K., Belting, H.-G. & Driever, W. Zebrafish pou5f1/pou2, homolog of mammalian Oct4, functions in the endoderm specification cascade. *Curr. Biol.* **14**, 48–55 (2004).
22. Ulianov, S. V. *et al.* Suppression of liquid-liquid phase separation by 1,6-hexanediol partially compromises the 3D genome organization in living cells. *Nucleic Acids Res.* **49**, 10524–10541 (2021).
23. Afgan, E. *et al.* The Galaxy platform for accessible, reproducible and collaborative biomedical analyses: 2018 update. *Nucleic Acids Res.* **46**, W537–W544 (2018).
24. Love, M. I., Huber, W. & Anders, S. Moderated estimation of fold change and dispersion for RNA-seq data with DESeq2. *Genome Biol.* **15**, 550 (2014).
25. Hiller, M. *et al.* Computational methods to detect conserved non-genic elements in phylogenetically isolated genomes: application to zebrafish. *Nucleic Acids Res.* **41**, e151 (2013).
26. Lift Genome Annotations. https://www.genome.ucsc.edu/cgi-bin/hgLiftOver.
27. Ramírez, F. *et al.* deepTools2: a next generation web server for deep-sequencing data analysis. *Nucleic Acids Res.* **44**, W160–5 (2016).

28. Li, H. Aligning sequence reads, clone sequences and assembly contigs with BWA-MEM. *arXiv [q-bio.GN]* (2013).

29. Open2C *et al.* Pairtools: From sequencing data to chromosome contacts. *PLoS Comput. Biol.* **20**, e1012164 (2024).

30. Kent, W. J. *et al.* The human genome browser at UCSC. *Genome Res.* **12**, 996–1006 (2002).

31. Abdennur, N. & Mirny, L. A. Cooler: scalable storage for Hi-C data and other genomically labeled arrays. *Bioinformatics* **36**, 311–316 (2020).

32. Imakaev, M. *et al.* Iterative correction of Hi-C data reveals hallmarks of chromosome organization. *Nat. Methods* **9**, 999–1003 (2012).

33. Barrett, T. *et al.* NCBI GEO: archive for functional genomics data sets--update. *Nucleic Acids Res.* **41**, D991–5 (2013).

34. Kaaij, L. J. T., van der Weide, R. H., Ketting, R. F. & de Wit, E. Systemic Loss and Gain of Chromatin Architecture throughout Zebrafish Development. *Cell Rep.* **24**, 1–10.e4 (2018).

35. Wike, C. L. *et al.* Chromatin architecture transitions from zebrafish sperm through early embryogenesis. *Genome Res.* **31**, 981–994 (2021).

36. Foster, E. D. & Deardorff, A. Open Science Framework (OSF). *J. Med. Libr. Assoc.* **105**, 203 (2017).

37. Llc, R. G. Reservoir. https://resgen.io/.

38. Open2C *et al.* Cooltools: Enabling high-resolution Hi-C analysis in Python. *PLoS Comput. Biol.* **20**, e1012067 (2024).

39. Yang, T. *et al.* HiCRep: assessing the reproducibility of Hi-C data using a stratum-adjusted correlation coefficient. *Genome Res.* **27**, 1939–1949 (2017).

40. Pedregosa, F. *et al.* Scikit-learn: Machine Learning in Python. *arXiv [cs.LG]* (2012).

41. Flyamer, I. M., Illingworth, R. S. & Bickmore, W. A. Coolpup.py: versatile pile-up analysis of Hi-C data. *Bioinformatics* **36**, 2980–2985 (2020).

42. Lieberman-Aiden, E. *et al.* Comprehensive mapping of long-range interactions reveals folding principles of the human genome. *Science* **326**, 289–293 (2009).

43. Spracklin, G. *et al.* Diverse silent chromatin states modulate genome compartmentalization and

loop extrusion barriers. *Nat. Struct. Mol. Biol.* **30**, 38–51 (2023).

44. Du, Z. *et al.* Allelic reprogramming of 3D chromatin architecture during early mammalian development. *Nature* **547**, 232–235 (2017).
45. Siefert, J. C., Clowdus, E. A., Goins, D., Koren, A. & Sansam, C. L. Profiling DNA Replication Timing Using Zebrafish as an In Vivo Model System. *J. Vis. Exp.* (2018) doi:10.3791/57146.
46. Crane, E. *et al.* Condensin-driven remodelling of X chromosome topology during dosage compensation. *Nature* **523**, 240–244 (2015).
47. Abdennur, N. *et al.* Bioframe: operations on genomic intervals in Pandas dataframes. *Bioinformatics* **40**, (2024).
48. Abdennur, N. *Pybbi: Python Bindings to UCSC BigWig and BigBed Library*. (Github).
49. Ulianov, S. V. *et al.* Order and stochasticity in the folding of individual Drosophila genomes. *Nat. Commun.* **12**, 41 (2021).
50. Bykov, N. S., Sigalova, O. M., Gelfand, M. S. & Galitsyna, A. A. HiChew: a Tool for TAD Clustering in Embryogenesis. in *Bioinformatics Research and Applications* 381–388 (Springer International Publishing, 2020).
51. Scharr, H. Optimal operators in digital image processing. (2000).
52. Scharr, H. Optimal Filters for Extended Optical Flow. in *Complex Motion* 14–29 (Springer Berlin Heidelberg, 2007).
53. Li, C. H. & Tam, P. K. S. An iterative algorithm for minimum cross entropy thresholding. *Pattern Recognit. Lett.* **19**, 771–776 (1998).
54. van der Walt, S. *et al.* scikit-image: image processing in Python. *PeerJ* **2**, e453 (2014).
55. Matthey-Doret, C. *et al.* Computer vision for pattern detection in chromosome contact maps. *Nat. Commun.* **11**, 5795 (2020).
56. Leichsenring, M., Maes, J., Mössner, R., Driever, W. & Onichtchouk, D. Pou5f1 transcription factor controls zygotic gene activation in vertebrates. *Science* **341**, 1005–1009 (2013).
57. Xu, C. *et al.* Nanog-like regulates endoderm formation through the Mxtx2-Nodal pathway. *Dev. Cell* **22**, 625–638 (2012).
58. Ewels, P. A. *et al.* The nf-core framework for community-curated bioinformatics pipelines. *Nat.*

Biotechnol. **38**, 276–278 (2020).

59. Tan, H., Onichtchouk, D. & Winata, C. DANIO-CODE: Toward an Encyclopedia of DNA Elements in Zebrafish. *Zebrafish* **13**, 54–60 (2016).
60. Castro-Mondragon, J. A. *et al.* JASPAR 2022: the 9th release of the open-access database of transcription factor binding profiles. *Nucleic Acids Res.* **50**, D165–D173 (2022).
61. *JASPAR-UCSC-Tracks: Code and Data Used to Create the JASPAR UCSC Genome Browser Tracks Data Hub*. (Github).
62. Patel, H., Wang, C. & Ewels, P. nf-core/chipseq: nf-core/chipseq v1. 2.2—Rusty Mole. *Zenodo https://doi. org/10.5281/zenodo*.
63. Eastman, P. *et al.* OpenMM 7: Rapid development of high performance algorithms for molecular dynamics. *PLoS Comput. Biol.* **13**, e1005659 (2017).
64. *Polychrom: Polymer Simulations of Chromosomes and Generating 'in Silico' Hi-C Maps*. (Github).
65. Goloborodko, A., Marko, J. F. & Mirny, L. A. Chromosome Compaction by Active Loop Extrusion. *Biophys. J.* **110**, 2162–2168 (2016).
66. Goloborodko, A., Imakaev, M. V., Marko, J. F. & Mirny, L. Compaction and segregation of sister chromatids via active loop extrusion. *Elife* **5**, (2016).
67. Fudenberg, G. *et al.* Formation of Chromosomal Domains by Loop Extrusion. *Cell Rep.* **15**, 2038–2049 (2016).
68. Fudenberg, G., Abdennur, N., Imakaev, M., Goloborodko, A. & Mirny, L. A. Emerging Evidence of Chromosome Folding by Loop Extrusion. *Cold Spring Harb. Symp. Quant. Biol.* **82**, 45–55 (2017).
69. Banigan, E. J. *et al.* Transcription shapes 3D chromatin organization by interacting with loop extrusion. *Proc. Natl. Acad. Sci. U. S. A.* **120**, e2210480120 (2023).
70. Mölder, F. *et al.* Sustainable data analysis with Snakemake. *F1000Res.* **10**, 33 (2021).
71. Rao, S. S. P. *et al.* A 3D map of the human genome at kilobase resolution reveals principles of chromatin looping. *Cell* **159**, 1665–1680 (2014).
72. Hoencamp, C. *et al.* 3D genomics across the tree of life reveals condensin II as a determinant of

architecture type. *Science* **372**, 984–989 (2021).

73. Rabl, C. Über Zelltheilung. Morphologisches Jahrbuch. 10. (1885).

74. Wang, X., Brandão, H. B., Le, T. B. K., Laub, M. T. & Rudner, D. Z. *Bacillus subtilis* SMC complexes juxtapose chromosome arms as they travel from origin to terminus. *Science* **355**, 524–527 (2017).

75. Brandão, H. B., Ren, Z., Karaboja, X., Mirny, L. A. & Wang, X. DNA-loop-extruding SMC complexes can traverse one another in vivo. *Nat. Struct. Mol. Biol.* **28**, 642–651 (2021).

76. Wang, X. *et al.* Condensin promotes the juxtaposition of DNA flanking its loading site in Bacillus subtilis. *Genes Dev.* **29**, 1661–1675 (2015).

77. Piazza, A. *et al.* Cohesin regulates homology search during recombinational DNA repair. *Nat. Cell Biol.* **23**, 1176–1186 (2021).

78. Liu, N. Q. *et al.* Extrusion fountains are restricted by WAPL-dependent cohesin release and CTCF barriers. *Nucleic Acids Res.* **53**, gkaf549 (2025).

79. Lüthi, B. N. *et al.* Cohesin forms fountains at active enhancers in C. elegans. *Genomics* (2023).

80. Kim, J., Wang, H. & Ercan, S. Cohesin organizes 3D DNA contacts surrounding active enhancers in C. elegans. *Genome Res.* **35**, 1108–1123 (2025).

81. Shao, W. *et al.* The jet-like chromatin structure defines active secondary metabolism in fungi. *Nucleic Acids Res.* **52**, 4906–4921 (2024).

82. Wang, D. *et al.* Promoter capture Hi-C identifies promoter-related loops and fountain structures in Arabidopsis. *Genome Biol.* **25**, 324 (2024).

83. Raney, B. J. *et al.* The UCSC Genome Browser database: 2024 update. *Nucleic Acids Res.* **52**, D1082–D1088 (2024).

84. Kruse, K. *et al.* Transposable elements drive reorganisation of 3D chromatin during early embryogenesis. *bioRxiv* 523712 (2019) doi:10.1101/523712.

85. Hsieh, T.-H. S. *et al.* Resolving the 3D Landscape of Transcription-Linked Mammalian Chromatin Folding. *Mol. Cell* **78**, 539–553.e8 (2020).

86. Goychuk, A., Kannan, D., Chakraborty, A. K. & Kardar, M. Polymer folding through active processes recreates features of genome organization. *Proc. Natl. Acad. Sci. U. S. A.* **120**,

e2221726120 (2023).

87. Brahmachari, S., Markovich, T., MacKintosh, F. C. & Onuchic, J. N. Temporally correlated active forces drive segregation and enhanced dynamics in chromosome polymers. *PRX Life* **2**, (2024).

88. Nuebler, J., Fudenberg, G., Imakaev, M., Abdennur, N. & Mirny, L. A. Chromatin organization by an interplay of loop extrusion and compartmental segregation. *Proc. Natl. Acad. Sci. U. S. A.* **115**, E6697–E6706 (2018).

89. Flyamer, I. M. *et al.* Single-nucleus Hi-C reveals unique chromatin reorganization at oocyte-to-zygote transition. *Nature* **544**, 110–114 (2017).

90. Rao, S. S. P. *et al.* Cohesin Loss Eliminates All Loop Domains. *Cell* **171**, 305–320.e24 (2017).

91. Niu, L. *et al.* Three-dimensional folding dynamics of the Xenopus tropicalis genome. *Nat. Genet.* **53**, 1075–1087 (2021).
