# Supplementary material for: Extrusion fountains are hallmarks of chromosome organization emerging upon zygotic genome activation
Source: Nat Commun. 2026 Feb 14;17:2787. doi: 10.1038/s41467-026-69105-9 (PMC13018191; doi:10.1038/s41467-026-69105-9)
Supplement: Supplementary file 2 — Description of Additional Supplementary Files [file 41467_2026_69105_MOESM2_ESM.pdf]

Description of Additional Supplementary Files

Supplementary Data 1. List of zebrafish fountains

Supplementary Data 2. Frog (Xenopus tropicalis) and medaka fish (Oryzias latipes) fountains

Supplementary Data 3. Zebrafish TAD boundaries

Supplementary Data 4. Zebrafish initiation zones

Supplementary Data 5. Fountains group assignment based on chromatin accessibility change in mutants

Supplementary Data 6. Detailed list of the sequencing datasets
