# Supplementary material for: Extrusion fountains are hallmarks of chromosome organization emerging upon zygotic genome activation
Source: Nat Commun. 2026 Feb 14;17:2787. doi: 10.1038/s41467-026-69105-9 (PMC13018191; doi:10.1038/s41467-026-69105-9)
Supplement: Supplementary file 9 — Reporting Summary [file 41467_2026_69105_MOESM9_ESM.pdf]

nature portfolio

Corresponding author(s): Galitsyna, Mirny, Onichtchouk

Last updated by author(s): Dec 23, 2025

Reporting Summary

Nature Portfolio wishes to improve the reproducibility of the work that we publish. This form provides structure for consistency and transparency in reporting. For further information on Nature Portfolio policies, see our Editorial Policies and the Editorial Policy Checklist.

Statistics

For all statistical analyses, confirm that the following items are present in the figure legend, table legend, main text, or Methods section.

n/a	Confirmed	
☐	☒	The exact sample size (*n*) for each experimental group/condition, given as a discrete number and unit of measurement
☐	☒	A statement on whether measurements were taken from distinct samples or whether the same sample was measured repeatedly
☐	☒	The statistical test(s) used AND whether they are one- or two-sided<br>*Only common tests should be described solely by name; describe more complex techniques in the Methods section.*
☐	☒	A description of all covariates tested
☐	☒	A description of any assumptions or corrections, such as tests of normality and adjustment for multiple comparisons
☐	☒	A full description of the statistical parameters including central tendency (e.g. means) or other basic estimates (e.g. regression coefficient) AND variation (e.g. standard deviation) or associated estimates of uncertainty (e.g. confidence intervals)
☐	☒	For null hypothesis testing, the test statistic (e.g. *F*, *t*, *r*) with confidence intervals, effect sizes, degrees of freedom and *P* value noted<br>*Give P values as exact values whenever suitable.*
☒	☐	For Bayesian analysis, information on the choice of priors and Markov chain Monte Carlo settings
☒	☐	For hierarchical and complex designs, identification of the appropriate level for tests and full reporting of outcomes
☐	☒	Estimates of effect sizes (e.g. Cohen's *d*, Pearson's *r*), indicating how they were calculated

Our web collection on statistics for biologists contains articles on many of the points above.

Software and code

Policy information about availability of computer code

Data collection

no software was used

Data analysis

Python 3.12
bwa-mem v0.7.17-r1188:
Li (2013). bwa-mem: Aligning sequence reads, clone sequences and assembly contigs with BWA-MEM. https://bio-bwa.sourceforge.net/bwa.shtml
cooler 0.10.4 for data reading, 0.8.10 for data storage:
Abdennur & Mirny (2019) cooler: scalable storage for Hi-C data and other genomically labeled arrays. https://cooler.readthedocs.io/en/latest/index.html
pairtools 1.1.3:
Open2C et al. (2023) Pairtools: from sequencing data to chromosome contacts. https://pairtools.readthedocs.io/en/latest/
cooltools 0.7.1 and 0.5.4:
Open2C et al. (2022) Cooltools: enabling high-resolution Hi-C analysis in Python. https://cooltools.readthedocs.io/en/latest/
HiChew 0.0:
Bykov et al. (2018) HiChew: a Tool for TAD Clustering in Embryogenesis. https://github.com/encent/hichew
Polychrom 0.1.1:
Imakaev (2019) polychrom: Polymer simulations of chromosomes and generating ‘in silico’ Hi-C maps. https://zenodo.org/record/3579473
HiGlass via Resgen (resgen.io) online server:
Kerpedjiev (2018) HiGlass: web-based visual exploration and analysis of genome interaction maps. https://resgen.io/
JASPAR whole-genome PWMScan-based motif scanner:
Fornes (2021) JASPAR UCSC tracks: whole-genome motif calling. https://github.com/wassermanlab/JASPAR-UCSC-tracks

chipseq-nf v 1.2.1:
Ewels et al. (2020) nf-core/chipseq: The nf-core framework for community-curated bioinformatics pipelines. https://nf-co.re/chipseq
sklearn 1.7.2:
van der Walt (2014) scikit-learn: image processing in Python. https://scikit-learn.org/
OpenMM 8.0:
Eastman et al. (2023) OpenMM 8: Molecular Dynamics Simulation with Machine Learning Potentials. J. Phys. Chem. B 128(1), pp. 109-116, 2023
liftover standard version:
Hinrichs et al. (2006) The UCSC Genome Browser Database: update 2006. Nucleic Acids Res. 34(Database issue):D590-8.
pybbi 0.4.2:
Abdennur (2025) https://github.com/nvictus/pybbi
bioframe 0.8.0:
Open2C et al. (2024) Bioframe: Operations on Genomic Intervals in Pandas Dataframes. https://bioframe.readthedocs.io/en/latest/
GREAT (Genomic Regions Enrichment of Annotations Tool) 2.5.4:
McLean et al. (2016) GREAT improves functional interpretation of cis-regulatory regions. Nature Biotechnology May 2;28(5):495–501. doi: 10.1038/nbt.1630

Bed Tools Quinlan and Hall,2010 BED Tools in usegalaxy.eu
Bowtie2 Langmead and Salzberg, 2012 Bowtie2 in usegalaxy.eu
DeepTools2 Ramirez et al., 2016 deepTools in usegalaxy.eu
DESeq2 Love et al., 2014 DESeq2 in usegalaxy.eu
FeatureCounts Liao et al., 2014 featureCounts in usegalaxy.eu
Galaxy server Afgan et al., 2018 https://usegalaxy.eu/
k-means clustering algorithm Ramirez et al., 2016 Available option in plotheatmap in deepTools2 in usegalaxy.eu
MACS2 Ferg et al., 2007 MACS2 callpeak in usegalaxy.eu
RNA Star Dobin et al., 2013 RNA Star in usegalaxy.eu
RNA-sense https://bioconductor.org/packages/release/bioc/html/RNAsense.html
geecee utility to calculate fractional GC content of nucleic acid sequences in usegalaxy.eu
R packages:

ggplot2 :
H. Wickham. ggplot2: Elegant Graphics for Data Analysis. Springer-Verlag New York, 2016.
dplyr:
Hadley Wickham, Romain FranÁois, Lionel Henry and Kirill M¸ller (2020). dplyr: A Grammar of Data Manipulation. R package version 1.0.2. https://CRAN.R-project.org/package=dplyr
eulerr:
Larsson J (2020). _eulerr: Area-Proportional Euler and Venn Diagrams with Ellipses_. R package version 6.1.0, <URL: https://cran.r-project.org/package=eulerr>.
NBPSeq:
Yanming Di, Daniel W Schafer, with contributions from Jason S Cumbie and Jeff H Chang. (2014). NBPSeq: Negative Binomial Models for RNA-Sequencing Data. R package version 0.3.0. https://CRAN.R-project.org/package=NBPSeq
qvalue:
John D. Storey, Andrew J. Bass, Alan Dabney and David Robinson (2019). qvalue: Q-value estimation for false discovery rate control. R package version 2.16.0. http://github.com/jdstorey/qvalue
SummarizedExperiment:
Martin Morgan, Valerie Obenchain, Jim Hester and HervÈ PagËs (2019). SummarizedExperiment: SummarizedExperiment container. R package version 1.14.1.
reshape2:
Hadley Wickham (2007). Reshaping Data with the reshape Package. Journal of Statistical Software, 21(12), 1-20. URL http://www.jstatsoft.org/v21/i12/.
tidyverse:
Wickham et al., (2019). Welcome to the tidyverse. Journal of Open Source Software, 4(43), 1686, https://doi.org/10.21105/joss.01686
matrixStats:
Henrik Bengtsson (2020). matrixStats: Functions that Apply to Rows and Columns of Matrices (and to Vectors). R package version 0.57.0. https://CRAN.R-project.org/package=matrixStats
hrbrthemes:
Bob Rudis (2020). hrbrthemes: Additional Themes, Theme Components and Utilities for 'ggplot2'. R package version 0.8.0. https://CRAN.R-project.org/package=hrbrthemes
viridis:
Simon Garnier (2018). viridis: Default Color Maps from 'matplotlib'. R package version 0.5.1. https://CRAN.R-project.org/package=viridis
parallel:
R Core Team (2019). R: A language and environment for statistical computing. R Foundation for Statistical Computing, Vienna, Austria. URL https://www.R-project.org/.
stats:
R Core Team (2019). R: A language and environment for statistical computing. R Foundation for Statistical Computing, Vienna, Austria. URL https://www.R-project.org/.
forcats:
Hadley Wickham (2020). forcats: Tools for Working with Categorical Variables (Factors). R package version 0.5.0. https://CRAN.R-project.org/package=forcats

For manuscripts utilizing custom algorithms or software that are central to the research but not yet described in published literature, software must be made available to editors and reviewers. We strongly encourage code deposition in a community repository (e.g. GitHub). See the Nature Portfolio guidelines for submitting code & software for further information.

Data

Policy information about availability of data

All manuscripts must include a data availability statement. This statement should provide the following information, where applicable:

- Accession codes, unique identifiers, or web links for publicly available datasets
- A description of any restrictions on data availability
- For clinical datasets or third party data, please ensure that the statement adheres to our policy

Raw and processed Hi-C data for zebrafish embryogenesis is available at GEO at https://www.ncbi.nlm.nih.gov/geo/query/acc.cgi?acc=GSE195609. The list of zebrafish fountains is available in Supplementary Dataset 1, Xenopus and medaka fountains in Supplementary Dataset 2, zebrafish TAD boundaries in Supplementary Dataset 3, zebrafish initiation zones at 4.3 hpf in Supplementary Dataset 4, and fountains group assignment based on chromatin accessibility change in mutants is available in Supplementary Dataset 5. The detailed list of the sequencing datasets is available in Supplementary Dataset 6. Additional datasets (including intermediary processed files) are available at OSF at https://osf.io/mt4vf. Interactive HiGlass views are available at Resgen at https://resgen.io/galitsyna/Zebrafish_embryogenesis/. Source data are provided with this paper.

Research involving human participants, their data, or biological material

Policy information about studies with human participants or human data. See also policy information about sex, gender (identity/presentation), and sexual orientation and race, ethnicity and racism.

Reporting on sex and gender	N/A
Reporting on race, ethnicity, or other socially relevant groupings	N/A
Population characteristics	N/A
Recruitment	N/A
Ethics oversight	N/A

Note that full information on the approval of the study protocol must also be provided in the manuscript.

Field-specific reporting

Please select the one below that is the best fit for your research. If you are not sure, read the appropriate sections before making your selection.

☒ Life sciences ☐ Behavioural & social sciences ☐ Ecological, evolutionary & environmental sciences

For a reference copy of the document with all sections, see nature.com/documents/nr-reporting-summary-flat.pdf

Life sciences study design

All studies must disclose on these points even when the disclosure is negative.

Sample size	Sample sizes for Hi-C were selected based on the common practice in the field: 1-2 mln nuclei were obtained from 500 embryos and were enough to prepare the material for one sample. Apart of the experiments listed here, no experiments in the zebrafish embryos were performed in the manuscript.
Data exclusions	There were no data exclusions.
Replication	Hi-C experiments were in 4 replicates for the wild-type embryos 5.3 hpf and in 2 replicates for all other genotypes and stages. All the attempts of the replication were successful.
Randomization	The samples were allocated to the experimental groups by genotype.
Blinding	Blinding was not relevant for the study, because the genotype of the mutant zebrafish embryos can be easily recognized from their phenotypic appearance.

Reporting for specific materials, systems and methods

We require information from authors about some types of materials, experimental systems and methods used in many studies. Here, indicate whether each material, system or method listed is relevant to your study. If you are not sure if a list item applies to your research, read the appropriate section before selecting a response.

Materials & experimental systems

n/a	Involved in the study
☒	☐ Antibodies
☒	☐ Eukaryotic cell lines
☒	☐ Palaeontology and archaeology
☐	☒ Animals and other organisms
☒	☐ Clinical data
☒	☐ Dual use research of concern
☒	☐ Plants

Methods

n/a	Involved in the study
☒	☐ ChIP-seq
☒	☐ Flow cytometry
☒	☐ MRI-based neuroimaging

Animals and other research organisms

Policy information about studies involving animals; ARRIVE guidelines recommended for reporting animal research, and Sex and Gender in Research

Laboratory animals	Danio rerio (zebrafish), males and females 0-2 years old, strains: AB/TL, MZspg, MZsox19b, MZnanog, MZps, MZsn, MZpn, MZtriple
Wild animals	no wild animals were used
Reporting on sex	Fish were used for breeding only, 50 to 50 males and females
Field-collected samples	no field samples were collected
Ethics oversight	All experiments were performed in accordance with German Animal Protection Law (TierSchG) and European Convention on the Protection of Vertebrate Animals Used for Experimental and Other Scientific Purposes (Strasburg, 1986). The generation of double mutants was approved by the Ethics Committee for Animal Research of the Koltzov Institute of Developmental Biology RAS, protocol 26 from 14.02.2019

Note that full information on the approval of the study protocol must also be provided in the manuscript.

Plants

Seed stocks	no plants were used
Novel plant genotypes	-
Authentication	-
